# Supplementary material for: TweetyBERT: Automated parsing of birdsong through self-supervised machine learning
Source: Patterns (N Y). 2026 Mar 3;7(4):101491. doi: 10.1016/j.patter.2025.101491 (PMC13083638; doi:10.1016/j.patter.2025.101491)
Supplement: Document S1. Figures S1–S7 and Tables S1 and S2 [file mmc1.pdf]

Patterns, Volume *7*

Supplemental information

TweetyBERT: Automated parsing of birdsong through self-supervised machine learning

George Vengrovski, Miranda R. Hulsey-Vincent, Melissa A. Bemrose, and Timothy J. Gardner

Supplementary

Layer	Sublayer	V-Measure	Dim.
3	Feed Forward Output	0.8935	196
4	Intermediate Residual Stream	0.8890	196
3	Intermediate Residual Stream	0.8859	196
3	Attention Output	0.8843	196
3	Feed Forward Output GELU	0.8829	768
2	Feed Forward Output	0.8757	196
2	Attention Output	0.8729	196
2	Feed Forward Output GELU	0.8619	768
1	Attention Output	0.8557	196
4	Attention Output	0.8494	196
4	Feed Forward Output	0.8420	196
2	Intermediate Residual Stream	0.8405	196
1	Feed Forward Output GELU	0.8400	768
1	Feed Forward Output	0.8326	196
4	Feed Forward Output GELU	0.8136	768
1	Intermediate Residual Stream	0.7518	196

Table A

Sublayer	Avg. V-Measure	Dim.
Attention Output	0.8656	196
Feed Forward Output	0.8609	196
Feed Forward Output GELU	0.8496	768
Intermediate Residual Stream	0.8418	196

Table B

Layer	Avg. V-Measure
3	0.8866
2	0.8627
4	0.8485
1	0.8201

Table C

Supplementary Table 1 | V-measure scores for layer and sublayer embeddings of TweetyBERT evaluated using UMAP projections from three birds in the TweetyNET dataset. *Scores represent averages across these test birds.* ***(A)*** *V-measure scores for each layer-sublayer combination. The selected representation (Layer 3 Attention Output) ranks fourth among all combinations; however, the difference from higher-ranked combinations is minimal and likely within measurement noise.* ***(B)*** *Crucially, Attention Output is the highest-performing sublayer averaged across all layers, and* ***(C)*** *Layer 3 provides the strongest overall representations when averaged across all sublayers, supporting its selection for downstream analyses.*

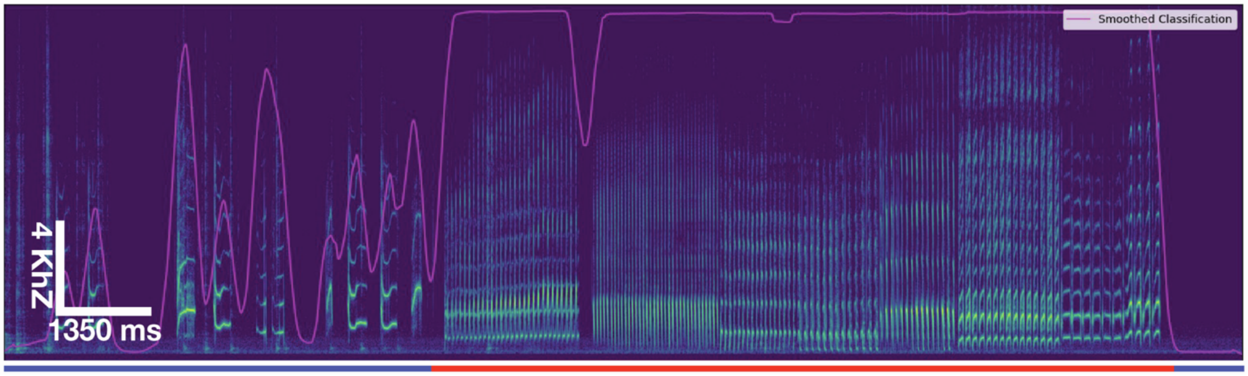

Supplementary Fig. 2 | Song Detector. *Spectrogram of a canary recording containing both song and non-song segments. The purple line represents the post-processed smoothed probabilities of each time bin being classified as canary song. The bottom bar displays the final Boolean classification of song (red) vs. non-song (blue) after post-processing. Notably, transient spikes around 1000 time bins—which are canary calls rather than song—are excluded from being labeled as song. The segmentation effectively distinguishes structured song from background noise, calls, and silent intervals, ensuring that only sustained vocal sequences are labeled as song. This figure represents a typical example of the classifier's performance across the dataset.*

The song detector used in this project is a lightweight variant of the TweetyNET architecture, designed to efficiently identify canary songs in spectrogram data. This model combines a convolutional front end with a bidirectional LSTM stack, maintaining a small hidden state size of 32 to optimize for speed and scalability. The song detector outputs a probability for each time bin, indicating the likelihood that the bin contains canary song. Ground truth labels are created by an expert human marking the start and stop of song. Spectrograms were generated using preprocessing methods previously described. During training, the model achieved a frame error rate (FER) of 2–3% on a test set containing 47 minutes of non-song and 27 minutes of song, demonstrating reliable detection of vocalizations versus background noise. The training was conducted with a learning rate of 3e-4, and the model was trained on 169 minutes of non-song and 107 minutes of song collected across a diverse set of recording conditions.

Post-processing plays a crucial role in refining the detector's output and ensuring reliable song annotations. While the raw FER is low, direct predictions can suffer from over segmentation, splitting continuous songs into fragments, or under segmentation, incorporating non-song noise into detected segments. The post-processing pipeline mitigates these issues by smoothing predictions, applying thresholds to eliminate short and noisy segments, and padding detected boundaries to ensure no valid song content is truncated. Although padding can introduce brief silences at segment edges, this tradeoff is acceptable as this has negligible performance impacts on the training of TweetyBERT.

It is important to note that the model's performance depends on the similarity between the training and deployment environments. Since the training data was manually annotated and collected in a consistent seasonal period and recording setup, the detector performs well within this context. However, deployment in different conditions may require retraining or fine-tuning to maintain accuracy.

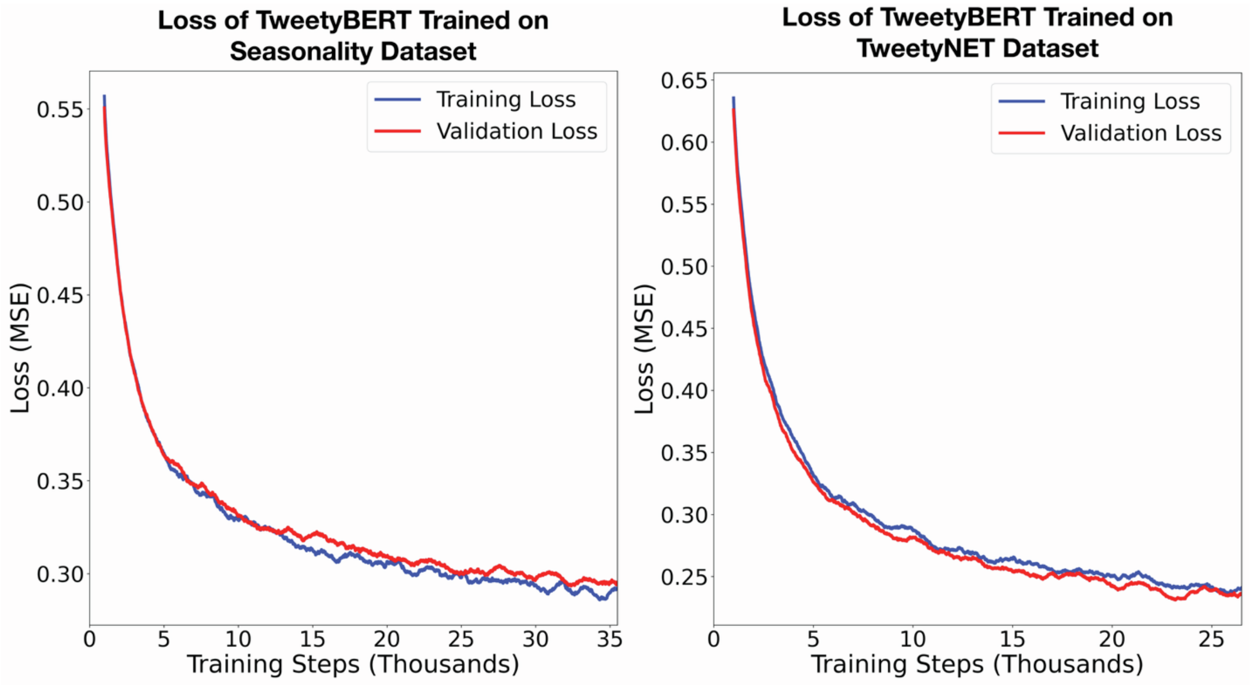

Supplementary Fig. 3 | TweetyBERT MSE Loss vs Training Steps. *Training and validation loss curves (mean squared error, MSE) for TweetyBERT trained separately on the Seasonality and TweetyNET datasets. Both training (blue) and validation (red) losses exhibit stable convergence, indicating effective self-supervised learning of masked spectrogram reconstructions. The higher final MSE observed for the Seasonality dataset likely reflects greater acoustic variability between breeding and non-breeding vocalizations, consistent with biologically driven seasonal vocal plasticity.*

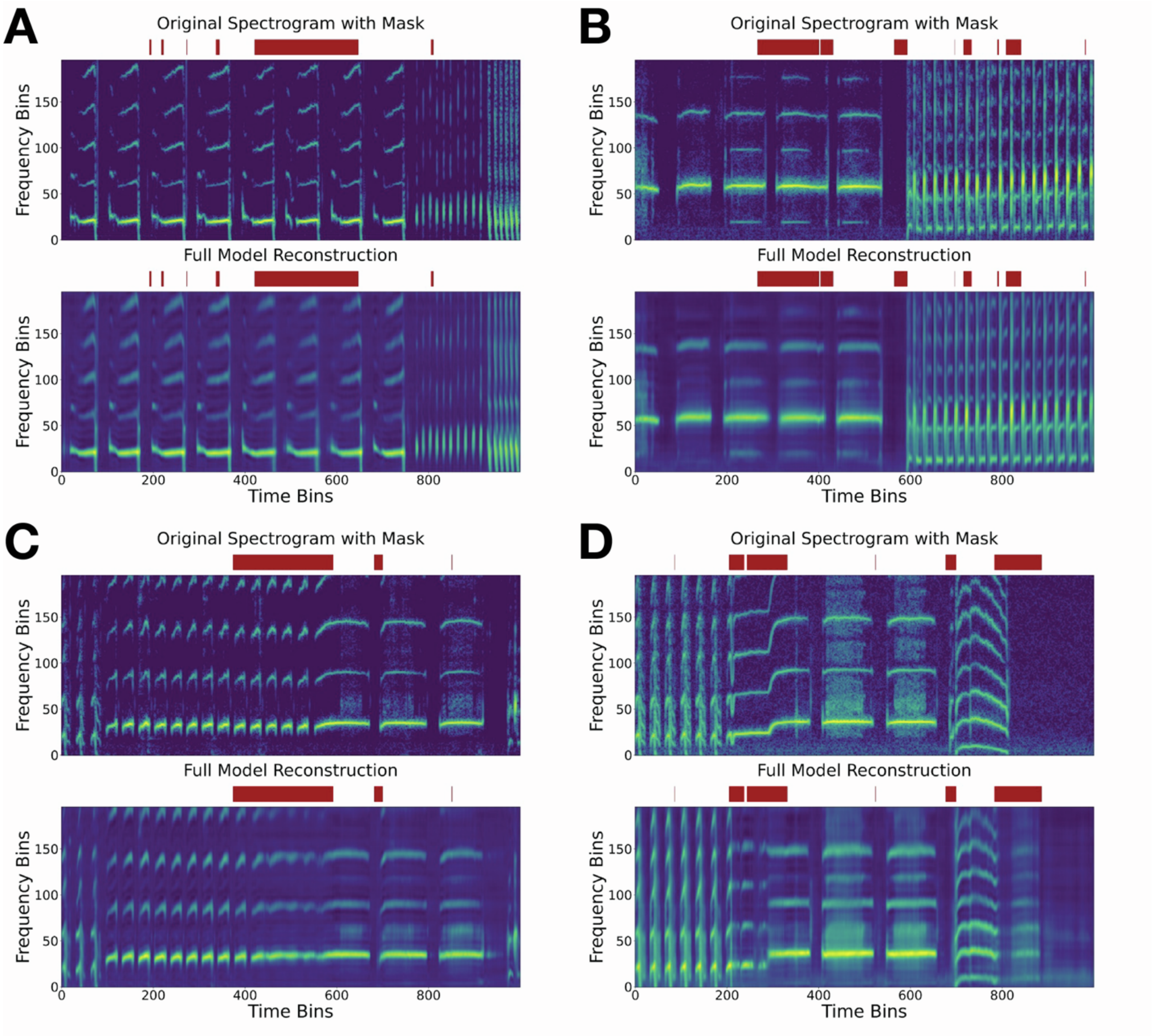

Supplementary Fig. 4 | Montage of masked spectrogram predictions from the TweetyBERT training phase. *Each panel consists of a masked spectrogram fed to TweetyBERT (top) and the model's predicted reconstruction (bottom). Red bars indicate masked regions that were not visible to the model during this prediction.* ***(A, B)*** *Depict accurate reconstructions of masked segments, demonstrating the model's capability to infer detailed acoustic structures from context.* ***(C, D)*** *Illustrate mild reconstruction errors where the model produces hybrid syllable morphologies by combining two syllable classes. These errors arise when critical contextual cues are obscured by masking, causing ambiguity in syllable prediction.*

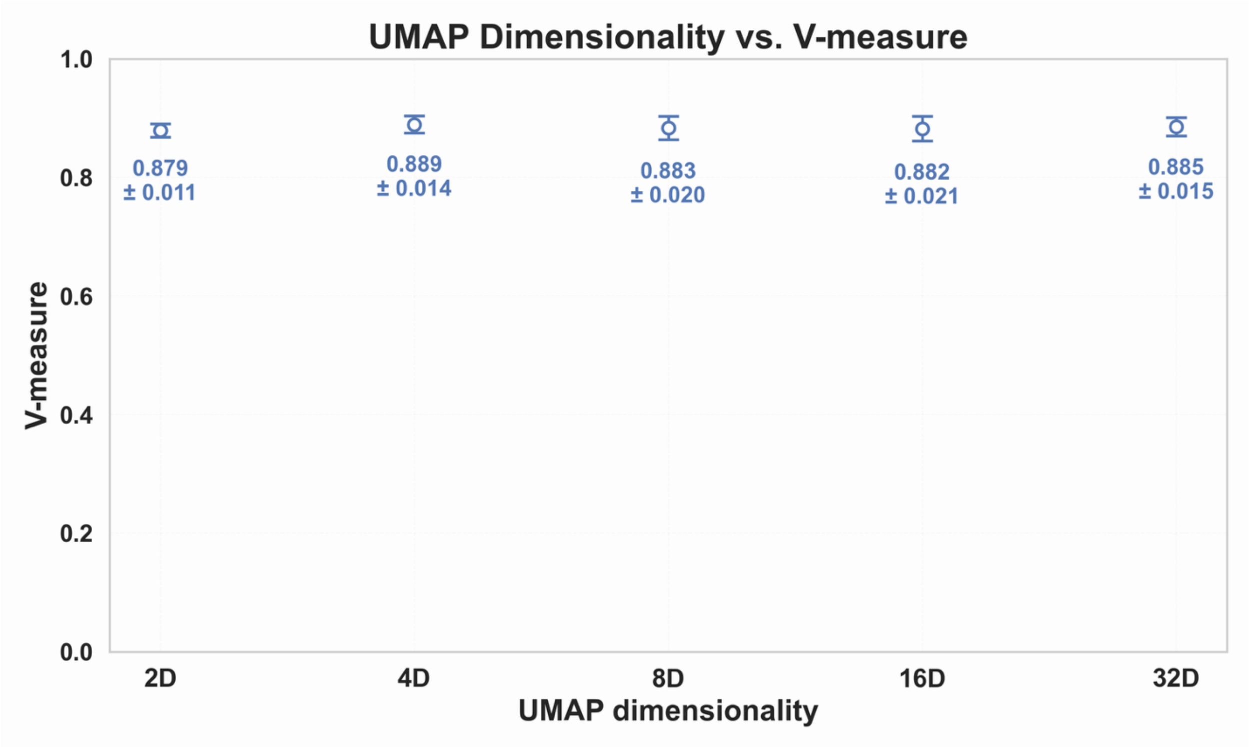

Supplementary Fig. 5 | V-Measure score as a function of UMAP Dimensionality. *The points summarize V-measure clustering scores obtained across 12 held-out folds of TweetyBERT latent representations. Boxes mark the standard deviation, and the filled circles indicate the fold-wise mean. It is apparent that the relationship between UMAP dimensionality and V-Measure score is weak.*

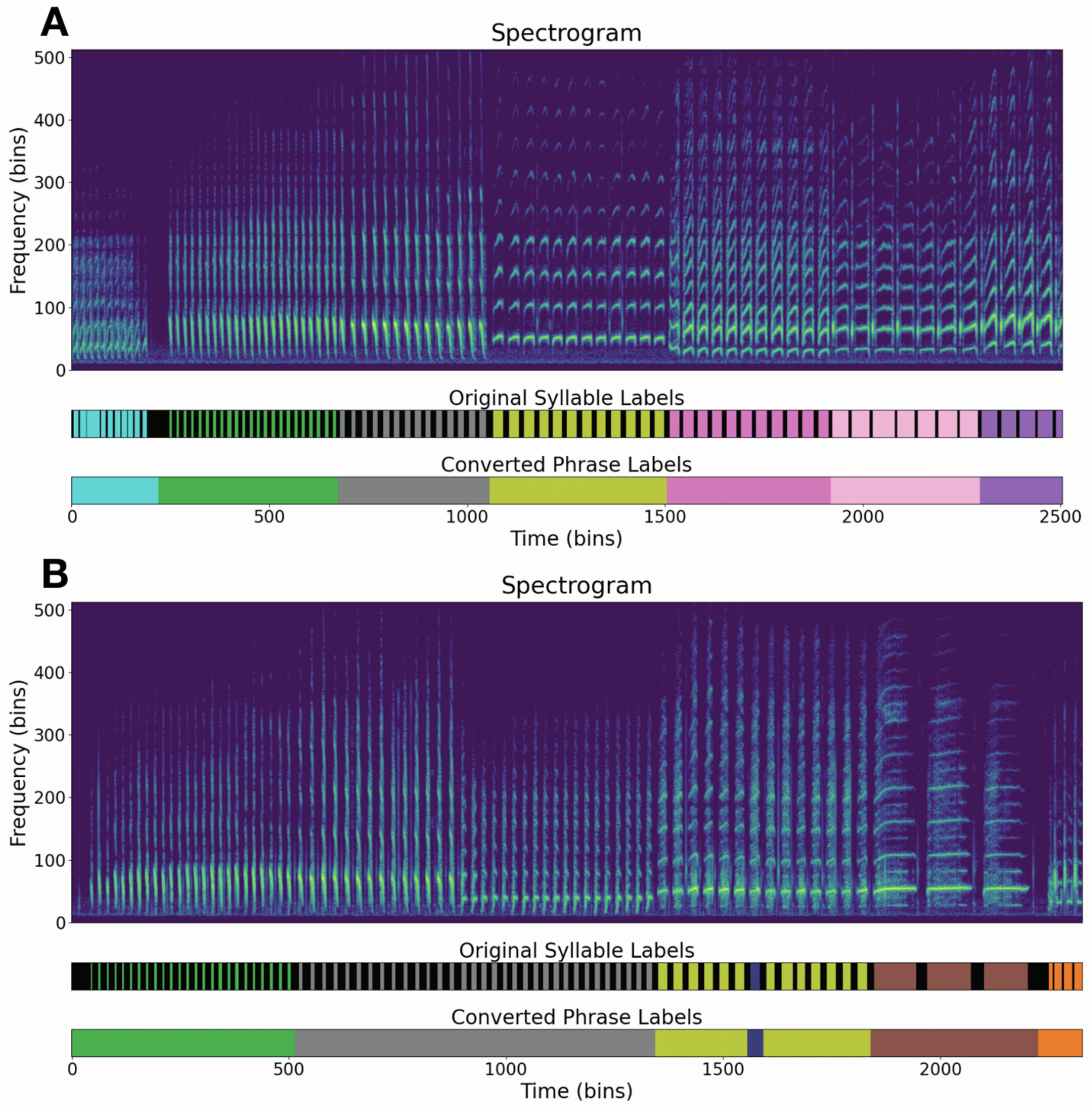

Supplementary Fig. 6 | Examples of conversion from syllable-level to phrase-level labels. ***(A, B)*** *Spectrograms of complete canary songs, each shown with original ground truth syllable-level annotations (top, silences in black) and their corresponding phrase-level labels after conversion (bottom). During conversion, silent intervals between syllables were reassigned to their nearest neighboring syllable labels. Panel* ***(B)*** *highlights two annotation errors in the original ground truth labels: a mislabeled syllable insertion (purple within yellow) and two distinct phrases incorrectly labeled as identical (gray).*

Smoothing Window (Time bins)	Phrase Entropy Correlation (r)	Phrase Duration Correlation (r)	V-Measure	FER (mapped)	FER (mapped and unmapped)
0	0.337	0.686	0.868	7.74%	15.98%
25	0.615	0.868	0.872	7.28%	15.59%
50	0.658	0.904	0.882	6.76%	15.12%
75	0.710	0.928	0.897	7.04%	15.73%
100	0.702	0.931	0.899	6.61%	15.65%
125	0.713	0.940	0.898	5.76%	14.92%
150	0.745	0.947	0.893	4.97%	14.51%
175	0.749	0.950	0.891	4.99%	14.52%
**200**	**0.771**	**0.957**	**0.888**	**4.34%**	**13.97%**
225	0.778	0.960	0.885	4.44%	14.06%
250	0.772	0.959	0.881	4.58%	14.18%
275	0.769	0.960	0.878	4.70%	14.29%
300	0.761	0.959	0.874	4.84%	14.42%
325	0.750	0.958	0.871	5.00%	14.53%
350	0.742	0.960	0.866	5.06%	14.66%
375	0.738	0.960	0.863	5.20%	14.78%
400	0.736	0.957	0.858	5.46%	14.94%
425	0.723	0.943	0.855	6.90%	16.24%
450	0.723	0.943	0.851	7.05%	16.42%
475	0.715	0.941	0.847	7.29%	16.63%
500	0.716	0.941	0.843	7.46%	16.87%

Supplementary Table 7 | Raw data evaluating the impact of smoothing window size on clustering performance. *Pearson correlations between HDBSCAN-derived and ground truth measures of phrase entropy and phrase duration, V-measure scores, and Frame Error Rates.*

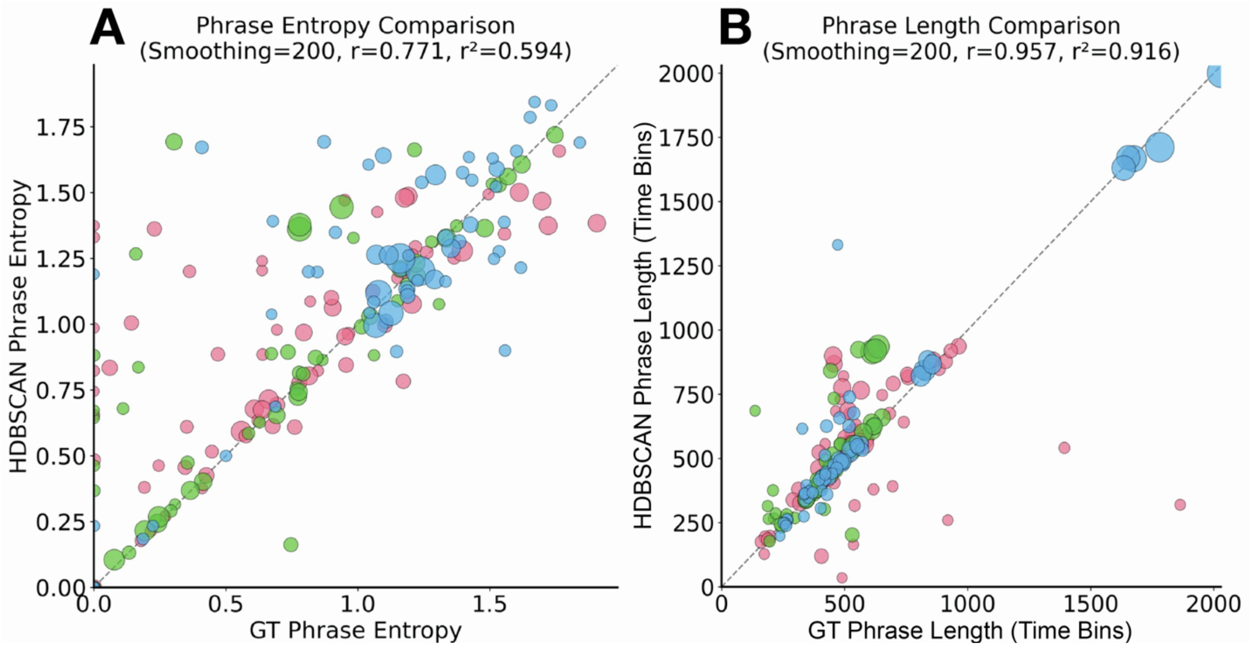

Supplementary Fig. 8 | Phrase entropy correlations for each individual phrase label across three birds. *Relationship between TweetyBERT-derived phrase metrics and human-derived ground truth (GT) metrics for three birds (colors represent individual birds).* ***(A)*** *Phrase entropy correlations* ***(B)*** *Phrase duration correlations. All analysis shown here were conducted with a 200 time bin smoothing window.*
