# Supplementary material for: TweetyBERT: Automated parsing of birdsong through self-supervised machine learning
Source: Patterns (N Y). 2026 Mar 3;7(4):101491. doi: 10.1016/j.patter.2025.101491 (PMC13083638; doi:10.1016/j.patter.2025.101491)
Supplement: Document S2. Article plus supplemental information [file mmc2.pdf]

Patterns

Article

TweetyBERT: Automated parsing of birdsong through self-supervised machine learning

Highlights

- TweetyBERT, a self-supervised transformer, learns canary song from spectrograms
- Dimensionally reducing self-attention states reveals structured syllable organization
- Clustering of the reduced space yields strong agreement with human annotations
- Embedding comparisons reveal vocal plasticity between spring and fall song

Authors

George Vengrovski,
Miranda R. Hulsey-Vincent,
Melissa A. Bemrose,
Timothy J. Gardner

Correspondence

timg@uoregon.edu

In brief

Parsing birdsong into behavioral units typically requires human-labeled data or pre-segmented audio. TweetyBERT, a self-supervised transformer, overcomes these limitations by learning directly from raw spectrograms. Operating at a 2.7 ms temporal resolution, the model preserves a one-to-one correspondence between input time bins and latent states. Applied to canary song, TweetyBERT autonomously discovers syllable-level representations that align closely with expert annotations, enabling large-scale automated labeling of vocal sequences with minimal human intervention.

Vengrovski et al., 2026, Patterns *7*, 101491
April 10, 2026 © 2025 Published by Elsevier Inc.
https://doi.org/10.1016/j.patter.2025.101491

CellPress

Patterns

CellPress
OPEN ACCESS

Article

TweetyBERT: Automated parsing of birdsong through self-supervised machine learning

George Vengrovski,[1,2] Miranda R. Hulsey-Vincent,[1,2] Melissa A. Bemrose,[2] and Timothy J. Gardner[1,2,3,*]
[1]Institute of Neuroscience and Department of Biology, University of Oregon, Eugene, OR, USA
[2]Phil and Penny Knight Campus for Accelerating Scientific Impact, University of Oregon, Eugene, OR, USA
[3]Lead contact
*Correspondence: timg@uoregon.edu
https://doi.org/10.1016/j.patter.2025.101491

THE BIGGER PICTURE The diversity of animal communication is staggering. With human speech representing only a small subset of vocal behavior on Earth, understanding the structure of nonhuman vocalizations remains an unexplored frontier. Beyond satisfying our curiosity about the vocal behaviors of our planet's many co-inhabitants, cataloging and analyzing animal communication could provide value for conservation efforts and understanding of the biosphere. Such information could serve as an early warning signal for loss of biodiversity as well as help map the habitation zones of critically endangered species. Songbirds represent a fascinating group of animal communicators because, like humans, they learn to vocalize, whereas most animals produce a fixed set of innate calls. Thus, parsing birdsong serves a dual purpose: it helps us understand how we acquire and produce speech, and it opens the door to systematically characterizing thousands of unique vocalizations in the wild, revealing the structure and variation in what these birds sing to one another. Parsing animal communication requires specific solutions tailored to each species. By combining large language models and massive quantities of unlabeled audio data, we may finally be positioned to characterize these vocalizations at scale. Here, we demonstrate how these breakthroughs can be leveraged in TweetyBERT, a self-supervised transformer model for parsing birdsong. This approach may generalize beyond canaries to diverse species, accelerating research into animal communication, vocal learning, and the neural mechanisms underlying vocal communication.

SUMMARY

Deep neural networks can be trained to parse animal vocalizations—serving to identify the units of communication and annotating sequences of vocalizations for subsequent statistical analysis. However, current methods rely on human-labeled data for training. The challenge of parsing animal vocalizations in a fully unsupervised manner remains an open problem. Addressing this challenge, we introduce TweetyBERT, a self-supervised transformer neural network developed for the analysis of birdsong. The model is trained to predict masked or hidden fragments of audio but is not exposed to human supervision or labels. Applied to canary song, TweetyBERT autonomously learns the behavioral units of song, such as notes, syllables, and phrases—capturing intricate acoustic and temporal patterns. This approach of developing self-supervised models specifically tailored to animal communication may significantly accelerate the analysis of unlabeled vocal data.

INTRODUCTION

Artificial intelligence offers great promise to enhance our understanding of animal vocalizations by enabling analysis of behavioral recordings at unprecedented scales.[1] Machine learning algorithms have already demonstrated substantial progress in classifying species-specific vocalizations, with applications such as Merlin Bird ID enabling species identification from environmental recordings.[2] However, the current challenge lies in developing methods that go beyond species identification to parse animal vocalizations into their fundamental behavioral units—notes, syllables, motifs, and bouts—that collectively structure complex vocal sequences.[3–6] Segmentation of vocalizations into these discrete behavioral units will enable detailed modeling of communication signals, linking specific vocal elements to behavioral contexts, emotional conditions,[7] or environmental stimuli. Achieving this level of analysis can help decode the "syntax" and "grammar" of animal communication, allowing

This is an open access article under the CC BY license (http://creativecommons.org/licenses/by/4.0/).

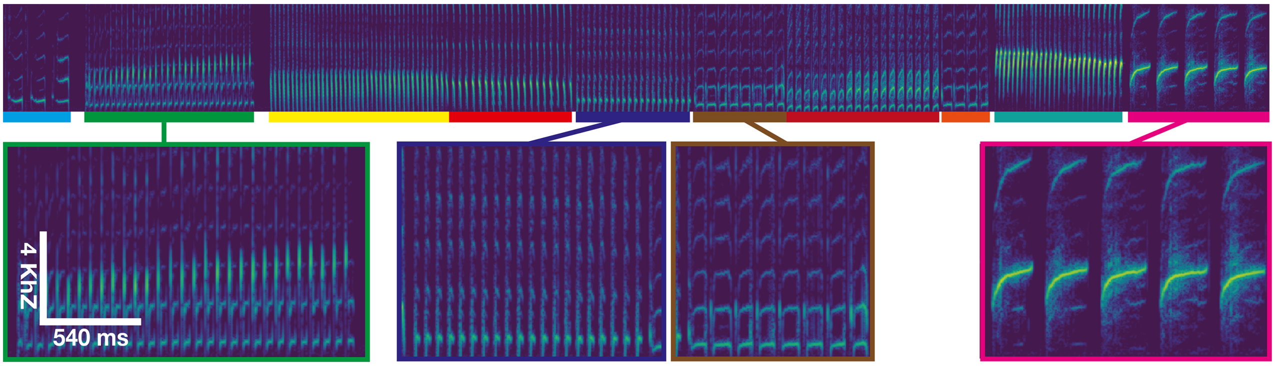

Figure 1. Typical canary song in spectrogram form

Canary song consists of stuttered syllables grouped in phrases (color bars) arranged sequentially.

for more detailed studies of the neural and cognitive mechanisms underlying vocal behavior.[8–12]

Supervised and semi-supervised methods for song segmentation have been pursued for many years[13–15] and are trained end-to-end to produce labels based on raw inputs, but these networks require human-labeled datasets for training.[6,16] For supervised models, the requirement for manual annotations severely restricts the scale and speed at which vocalization data can be analyzed. To circumvent these limitations, unsupervised methods seek to develop song representations directly from acoustic data without human annotation.[4,17,18] An essential step in this unsupervised parsing of animal vocal sequences involves creating a latent feature space (or embedding) in which acoustically similar sounds cluster together. Approaches established in the field include the use of variational autoencoders (VAEs) or standard autoencoders (AEs) or even direct dimensionality reduction techniques such as uniform manifold approximation and projection (UMAP) to generate representations that are then clustered. These methods have been shown to produce clusters that closely match human-defined categories of vocal units.[4,5,18]

However, these approaches typically take as input pre-segmented songs, and this segmentation process relies on human priors about where vocal units begin and end, and for some species, such as the canary, this segmentation is challenging and may require a human in the loop.[4,6] In the case of the canary and other species, song syllables can vary in duration from tens of milliseconds to hundreds of milliseconds, and existing methods require padding or otherwise extending the short syllables to match the timescale of the long syllables. Once padded, current unsupervised methods compress syllable forms into a latent space, using the same latent-space dimension for short and long syllables. While this pre-segmentation analysis can work well in many cases, such as zebra finch,[19] here we ask if a new class of unsupervised learning can discover the units of song with less human intervention—specifically, we are seeking an unsupervised method that will process song and discover the units of song directly without the steps of pre-segmentation and padding. For this task, we turn to the transformer architecture.

The transformer architecture was originally designed for natural language translation tasks, where it was shown to excel in modeling the complex temporal relationships among elements within long sequences.[20] This is enabled by self-attention, a mechanism that integrates information from all data points in a sequence during processing of each individual point. The architecture revolutionized natural language processing,[21–23] and permutations of the transformer architecture have successfully generalized beyond text-based tasks and are commonly applied to speech, vision, and multimodal information.[24–26]

The transformer architecture has made significant inroads in bioacoustics. With supervised fine-tuning, transformer models can detect and classify species within a recording.[3,27,28] Identification of individual marmosets from call sequences has also been reported.[29] Transformer encoder models pretrained on human or bioacoustics data create latent-space representations that can separate bird, bat, and monkey vocalizations with simple linear operations.[30–32] However, these approaches typically aggregate all the transformer sequence elements into a single representation or require fine-tuning for the classification of units of vocalization. To our knowledge, the fine-grained temporal structure of transformer latent spaces has not been leveraged for fully unsupervised analysis of birdsong sequences. This project demonstrates automated discovery of the units of a bird's song in a manner similar to how Wav2Vec2 developed internal representations of human phonemes through unsupervised analysis of raw speech.[33] While the models are closely related to prior human speech models, a key distinction is that we apply these models at much higher temporal resolution to capture the fast timescales of birdsong syllables.[34]

Here, we introduce TweetyBERT, a self-supervised, transformer-based neural network specifically designed for automated discovery of song structure. The model employs an order of magnitude greater temporal detail than typical human speech transformer models. To evaluate the model, we focus on a species that we have studied in detail before—the domestic canary—but we anticipate that it can be applied to other songbirds with minor adopted modifications (Figure 1). We demonstrate that TweetyBERT spontaneously learns representations of individual syllables as distinct elements within its latent embedding space. Remarkably, this structured representation emerges solely from a masked prediction task, without explicit supervision on syllable boundaries or categories. Furthermore, we show that TweetyBERT's latent space exhibits high consistency

CellPress
OPEN ACCESS

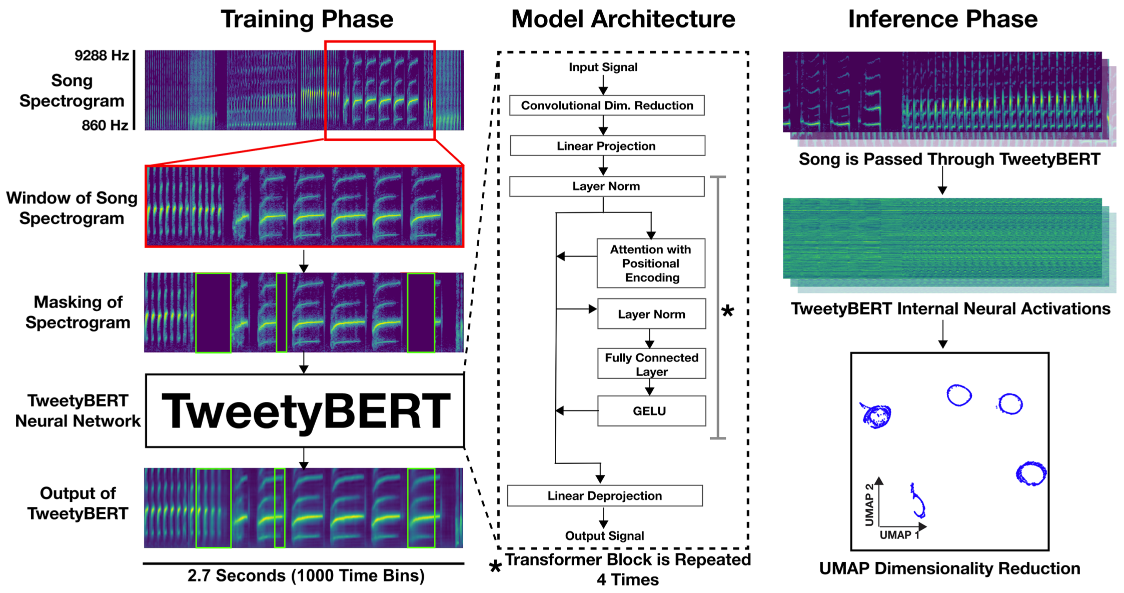

Figure 2. TweetyBERT architecture

Training phase: segments (2.7 s) of canary song spectrograms are masked and input to the TweetyBERT network, which learns to reconstruct masked spectrogram regions. Model architecture: TweetyBERT is a self-supervised transformer network operating directly on spectrogram inputs, combining a convolutional front-end (for local acoustic feature extraction) with a compact transformer backbone. Inference phase: song spectrograms are passed through the trained network, and the latent representations from the multi-head attention of the 3rd transformer block are extracted for analysis. The UMAP visualization shows that TweetyBERT's latent representations corresponding to distinct syllable types typically form elliptical trajectories in reduced dimensional space.

for individual canaries recorded at different times within the spring breeding season, but it also reveals significant variations between breeding season song and fall plastic song,[35,36] suggesting that the model will be useful for qualitative analysis of song structure beyond syllable identification. Whether through automated clustering of vocalizations or qualitative analysis of latent-space trajectories, self-supervised transformer models developed specifically for animal vocalizations will open new doors in the analysis of animal communication.

METHODS

Model overview

TweetyBERT is a compact spectrogram-based, self-supervised transformer network inspired by the architectures of Bidirectional Encoder Representations from Transformers (BERT) and Transformer Encoder Representations from Alteration (TERA).[21,37] The model operates directly on spectrograms without discretization or vector quantization, reducing both preprocessing complexity and hyperparameter dependencies. Both existing bioacoustics transformers and transformers designed for processing human speech typically aggregate temporal information into coarse sequence elements, where many audio samples or spectrogram time bins are represented as a single sequence element in the transformer's latent space[24,33,38]; instead, TweetyBERT preserves the input's full temporal resolution, maintaining a one-to-one correspondence between input time bins and latent states at every network layer. This design choice enables precise tracking of how the network processes each moment in the canary song.

TweetyBERT learns through pixelwise reconstruction of masked spectrogram regions, a training objective that encourages the network to develop internal representations of canary songs. As is typical in self-supervised learning, the reconstruction task itself is discarded after training, and instead, our primary insights come from analyzing the model's internal latent representations, not its output predictions (Figure 2, inference phase). TweetyBERT is not a foundation model but rather is designed to be pretrained on a group of birds that are representative of the recording conditions in the analysis set. Throughout the paper, we often refer to TweetyBERT's internal latent representations; when we do so, we are referencing the latent representation extracted from the multi-head self-attention layer of the 3rd transformer block, which contains features that show high correspondence with canary phrase-level identity (Table S1).

Spectrogram generation and preprocessing

Spectrogram generation parameters were adapted from the TweetyNET paper's established methodology.[6] Raw audio recordings were processed using a 5th-order elliptic high-pass filter (0.2 dB ripple, 40 dB stopband attenuation, and 500 Hz cutoff) to remove low-frequency noise that could interfere with song analysis. The short-time Fourier transform was then applied using a Hann window with a 1,024-point fast Fourier transform and 119-sample hop length, yielding spectrograms with a 2.7 ms hop size per time bin. Throughout this paper, the terms "frames," "time bins," and "points" all refer to these discrete 2.7 ms temporal units. This 2.7 ms resolution was chosen to capture the finest temporal structure in canary song—it is just shorter than the briefest inter-syllabic gaps, preventing frames from

straddling syllable boundaries.[6] Spectrogram inputs underwent several preprocessing steps. The frequency dimension was truncated to the range of [20, 216] frequency bins [860, 9,288 Hz], conservatively retaining the frequencies most relevant to canary vocalizations.[39] Also, each spectrogram was Z score normalized across the entire frequency-time matrix (not column-wise Z scoring but rather image-wise), stabilizing input distributions and improving training stability. Spectrogram generation was restricted to time intervals marked as containing songs by the song detector (Figure S2). Spectrogram processing was performed using Librosa[40] and SoundFile.[41]

Model architecture (TweetyBERT)

The model processes spectrograms spanning 196 frequency bins by 1,000 time bins, corresponding to 2.7 s of audio. Although a 2.7 s context window does not encompass entire canary songs, we avoided using longer segments, such as entire songs (up to 60 s long), due to the quadratic memory requirements inherent in transformer architectures. This segment length is similar to that used in prior supervised deep learning methods, where optimal segments were approximately 1 s long.[6] Our hyperparameter choices were guided by intuition and prior literature and should be regarded as preliminary—they were not optimized through ablations or hyperparameter comparisons.

The overall TweetyBERT architecture sequentially integrates the following components: (1) spectrogram masking, (2) a convolutional neural network (CNN) front-end for local feature extraction, (3) linear projection of CNN outputs into a transformer-compatible embedding space, (4) stacked transformer encoder blocks for modeling temporal dependencies and feature encoding, (5) linear projection back into spectrogram space, and (6) computation of a masked reconstruction loss (mean squared error [MSE]).

The convolutional front-end contains four convolutional layers (Conv1 with 32 channels and Conv2–Conv4 with 64 channels each), each followed by Gaussian Error Linear Unit (GELU) activation and max-pooling operations. Specifically, convolutional layers utilize a kernel size of 5×5 and padding of 2 and are followed by max-pooling layers with kernel sizes of (2,1) to reduce dimensionality selectively along the frequency axis while preserving temporal resolution. We made an explicit design choice to include a convolutional front-end prior to the transformer for several reasons: convolutional layers are a computationally cheap way to learn local spectral patterns without the memory-heavy attentional operations, many networks geared toward high-performance birdsong classification and detection rely on convolutional layers,[2,6,42] and there is precedent for combining convolutional operations with transformers to process audio and image data.[24,33,43]

The transformer encoder is the central component of TweetyBERT, consisting of four identical encoder blocks adapted from the original BERT architecture, incorporating enhancements to improve performance and training stability. Each encoder block comprises a multi-head self-attention layer with four attention heads (head dimension = 49, total embedding dimension = 196), followed by a feedforward neural network (FFN) with a hidden layer dimension of 768. The transformer blocks incorporate relative learned positional encodings, enabling the model to represent contextual relationships between time bins based on their relative positions rather than absolute locations, thus providing translation invariance across the sequence.[44] We further adapted the original BERT design by applying pre-layer normalization to improve gradient stability and training convergence and included GELU activation functions within the FFN layers to provide smooth nonlinear transformations.[45,46] Qualitatively, we found that these choices led to adequate training speed, convergence behavior, and overall stability. The complete TweetyBERT model (CNN front-end plus transformer encoder) contains approximately 2.5 million parameters.

Pretraining protocol and preprocessing

For pretraining TweetyBERT, the dataset was partitioned into an 80/20 train-test split at the song level, ensuring that entire songs were exclusively assigned to either the training or test set. This strategy prevented data leakage by ensuring the model never encountered fragments of the same song in both sets. Two distinct models were trained: one on the TweetyNET dataset, comprising ∼15 h of song for training and ∼4 h in a holdout set used for early stopping and downstream analysis, and another for seasonality analysis, trained with ∼20 h of song and a holdout set of ∼5 h. The TweetyNET dataset model was trained for 26,500 optimization steps, while the seasonality model underwent 35,500 optimization steps, with each optimization step corresponding to one batch (Figure S3).

During pretraining, we aimed to mitigate potentially trivial solutions to the masked prediction task that would hamper performance or generalizability. To do so, we wanted each training sample to be unique; that way, the model could not exploit the pitch of a syllable nor peculiarities in the syllables' positioning within a window to easily predict the pixels behind the mask. To achieve this, we had the data loader dynamically extract random 1,000 time-bin segments (∼2.7 s) from larger spectrograms. Additionally, random frequency shifts of ±50 bins (∼2 kHz) were applied to the spectrogram image during training, which, even though it introduces significant variation in frequency content beyond the natural frequency variability of adult canary song, is still consistent with prior bioacoustics audio models.[2,47] The shift operation was performed before the masking step, ensuring that inputs and targets share the same shifted reference frame. We used zero padding to fill the frequency axis positions that became vacant after applying the shift. For spectrograms shorter than 1,000 time bins, zero padding was applied along the temporal axis during pretraining to maintain consistent input dimensions across the batch size.

A masked prediction objective guided the model's self-supervised learning. We applied the masks before the convolutional front-end processed the spectrogram, preventing the model from cheating by exploiting convolutional receptive fields that might span between masked and unmasked regions. 25% of the input spectrogram was randomly occluded by masks of varying lengths, sampled uniformly from 0 to 250 time bins (0–675 ms), with each mask spanning all the frequency bins for the region (Figure S4). Masked regions were replaced with zeros. Our masking strategy followed the same full-frequency masking approach as TERA, but we applied a higher masking ratio.[37] We chose this higher ratio due to the stereotyped nature of canary song, which makes it more predictable than speech. We did

not perform mask ratio ablations, as this ratio proved effective for our purposes. To increase the per-training-step training speed, identical masks were applied across all batch elements per optimization step.

Loss, optimization, and early stopping

The training objective minimized an MSE loss, computed exclusively over the masked regions. Optimization was performed using the Adam optimizer with a learning rate of 3e−4 and a batch size of 42.[48] The training loop utilized mixed-precision training with torch.cuda.amp and GradScaler to reduce memory consumption and increase training speed.[49] Validation was conducted at regular intervals (every 500 steps), with the validation loss smoothed using a 1,000-step moving average to mitigate noise in performance evaluations.

An early stopping criterion was employed to ensure efficient convergence. Our early stopping scheme is conceptually based on the approach in TweetyNET but is more conservative.[6] Training terminated if the smoothed validation loss did not improve for eight consecutive validation checks. This mechanism prevented excessive overfitting and halted training once the model had converged.

Embedding generation and dimensionality reduction

To generate TweetyBERT embeddings, up to 1 million spectrogram time bins (∼45 min of song) were processed (Figures 3 and 7). The latent representation from the multi-head self-attention layer of the 3rd transformer block was the focus of subsequent analysis. This layer was chosen for its high V-measure scores (Table S1). Since the model preserves high temporal resolution everywhere, this yielded 1 million time latent representation vectors. These 196-dimensional (196D) activations were reduced to 2D via UMAP (cosine distance, 200 neighbors, minimum distance = 0.1, seed = 42), as higher dimensions yielded no benefit (Figure S5). UMAP parameters were chosen to prioritize clear separation between clusters, with the high neighbor count and moderate minimum distance promoting distinct, well-separated groupings in the embedding space. For spectrogram embeddings, we used the same UMAP parameters and directly applied dimensionality reduction to the Z scored individual frequency vectors of the spectrogram to enable direct comparison with the TweetyBERT embedding.

For temporal sequence analysis, clustering optimization, and V-measure calculations, only unseen songs from the holdout set were used for embedding generation, providing a measure of the model's generalization performance. We additionally used data "folds" because embedding the entire test set at once would exceed manageable data limits for a single UMAP. Breaking the data into multiple folds (ranging from 288,876 to 685,969 time bins, averaging ∼442,502) also allowed us to construct statistical measures (e.g., V-measure) across random dataset breakdowns for each bird, thus capturing the variability in performance within and between folds.

Syllable-to-phrase label conversion

When necessary, we converted the human syllable-level ground-truth labels from the TweetyNET dataset into phrase-level labels by replacing inter-syllabic silent bins with their nearest adjacent syllable label (Figure S6). In case of a tie, we took the syllable label earlier in the song. For non-song regions at the beginning or end of a file (i.e., where no bounding label was present on one side), the silence labels were replaced with the adjacent syllable label.

Clustering with HDBSCAN

TweetyBERT embeddings were clustered using the Hierarchical Density-Based Spatial Clustering of Applications with Noise (HDBSCAN) implementation in a prior study,[50] with a minimum sample of 1 and a minimum cluster size of 5,000. Given the large quantity of spectrogram time bins being processed through UMAP, we deliberately chose a conservative (large) minimum cluster size of 5,000 to avoid overclustering. We set the minimum sample parameter to 1 to ensure maximum point assignment to clusters (minimizing noise points). This represents a deliberate trade-off: the low minimum sample value makes the algorithm more permissive in forming clusters, but the high minimum cluster size ensures that only substantial groupings are identified. These HDBSCAN parameters could likely be improved, as we did not conduct an exhaustive parameter search given the exploratory nature of this work.

Temporal smoothing

After clustering with HDBSCAN, we applied a temporal smoothing algorithm to reduce transient fluctuations in cluster assignments. Specifically, for each labeled sequence of length n, we took a sliding window of length w around each position i and replaced the label at position i with the most frequent label in that window. In the event of a tie, the algorithm selected the label that appeared first in the window. At the start and end of sequences, window sizes were dynamically adjusted to fit the available data.

Replacing noise with nearest neighbor

HDBSCAN designates outlier points as noise. In practice, only a small fraction of time bins were labeled as noise by HDBSCAN. To preserve potentially meaningful data, we reassigned these noise-labeled points to the nearest valid cluster within the same sequence of labels. Specifically, for each noise-labeled bin, we searched leftward and rightward to locate the nearest labeled bins, measured the distance in time bins, and adopted the label of the nearest neighbor. If both neighbors were equidistant, we defaulted to the left label. In rare cases where neither side contained a valid label, the original noise label remained unchanged. This procedure was applied to the V-measure, correlation, and smoothing analyses.

V-measure

V-measure is a widely used metric for evaluating clustering performance, calculated as the harmonic mean of two complementary components: homogeneity, which assesses how well clusters contain members of a single ground-truth class, and completeness, which evaluates how well each ground-truth class maps to individual clusters (in our case, clusters derived from HDBSCAN).[51,52] The V-measure ranges from 0 to 1, where 1 indicates perfect clustering and 0 indicates complete disagreement between clusters and ground-truth labels.

Frame error rate

The frame error rate (FER), adapted from TweetyNET,[6] quantifies the percentage of time bins (2.7 ms spectrogram frames) in

which predicted labels disagree with ground-truth annotations, ranging from 0% (perfect classification) to 100% (complete disagreement). This provides a standardized measure of classification accuracy across both unsupervised clustering and supervised linear probe evaluations. We describe in detail how the FER is utilized for both unsupervised and linear probe analyses in the respective sections.

Mapping HDBSCAN labels to ground-truth labels

To map discovered labels to existing ground-truth labels, a shared-area matrix, M, was constructed such that each entry $M_{(i,j)}$ represented the number of frames in which ground-truth label i co-occurred with predicted label j. M was then column normalized, dividing each column by its total count to convert raw co-occurrence counts into proportions, ensuring that predicted clusters of different sizes are compared fairly. Subsequently, the Hungarian algorithm was applied to the normalized matrix, yielding an optimal one-to-one assignment pattern between ground-truth labels and predicted clusters that maximized diagonal alignment so that predicted labels were matched to ground-truth labels in a way that maximized co-occurrence.[53] This approach matches each ground-truth label exclusively to the single best-predicted cluster, inherently leaving some ground-truth or HDBSCAN labels without a match (depending on which one had more labels). These unmapped predicted clusters were designated as "unmatched" for evaluation purposes.

Phrase metrics

For each mapped pair of ground-truth and HDBSCAN-predicted phrase labels, we computed the following metrics for both human (ground-truth) and automated (predicted) annotations.

(1) Phrase transition entropy: for each phrase, we recorded how often it transitioned to each other phrase in the dataset. We then used Shannon entropy to measure the unpredictability of these transitions, averaging across all occurrences of the phrase. Higher entropy implies a more varied set of possible "next" phrases, whereas lower entropy indicates more predictable transitions.[12]
(2) Phrase duration: phrase duration was defined as the average length of uninterrupted time bins assigned to each phrase label. Specifically, for each labeled phrase, we identified continuous runs of that phrase in the data and computed their mean duration.

Weighted Pearson correlation

To measure how well the automated (predicted) values of entropy or phrase length match the same measures derived from ground-truth labels, we computed a weighted Pearson correlation. Each phrase received a weight proportional to its frequency of occurrence, ensuring that commonly used phrases had a greater influence on the correlation value.

Linear probe analysis

As an alternative to UMAP and HDBSCAN-based measures of model performance, we also employ linear probes, as described in the results section. The linear probe consisted of a single linear layer mapping 196D embeddings to phrase probabilities. For the pretrained and untrained conditions, the probe was applied to the multi-head self-attention layer from the 3rd transformer block, while for the fine-tuned condition, it was applied to TweetyBERT's final linear deprojection layer. For the spectrogram condition, the probe was applied directly to raw spectrogram features. The linear probe was trained on the training set using Adam optimization,[48] with learning rates of 1e−2 (linear probe, spectrogram, untrained) and 3e−4 (fine-tuned); these values were crucial for convergence. Mixed-precision training[49] and a batch size of 42 were used to reduce memory consumption and accelerate training. Frequency-shifting augmentation or masking was not applied during training or evaluation of the linear probe. Early stopping with a patience of six evaluation intervals (25 batches per interval) and a moving average of validation loss over 1,000 steps was implemented to stabilize training and prevent overfitting. Training was capped at 5,000 batches.

Accuracy was measured as the FER, calculated as the percentage of mismatched time bins between linear probe predictions and ground-truth labels. The FER was computed as the average across the three labeled birds from the TweetyNET holdout dataset. The pretrained and fine-tuned models shared the same TweetyBERT backbone, trained on the TweetyNET dataset, while the untrained model was initialized randomly.[54]

Seasonal embedding analysis

TweetyBERT embeddings were used to characterize seasonal differences in canary song structure across breeding and non-breeding seasons. For each bird, song data from approximately 1 million time bins were projected into a common 2D latent embedding space (UMAP coordinates). The resulting embeddings were divided equally into four distinct temporal groups: two groups from the breeding season and two groups from the non-breeding season.

Specifically, for canary 1, the recording dates for each group were as follows:

(1) Breeding season 1: May 28, 2024–May 29, 2024
(2) Breeding season 2: May 30, 2024–May 31, 2024
(3) Non-breeding season 1: September 13, 2024–September 21, 2024
(4) Non-breeding season 2: September 22, 2024–September 27, 2024

For canary 2, the corresponding dates were as follows:

(1) Breeding season 1: May 30, 2024–June 1, 2024
(2) Breeding season 2: June 2, 2024–June 3, 2024
(3) Non-breeding season 1: September 13, 2024–September 16, 2024
(4) Non-breeding season 2: September 17, 2024–September 20, 2024

The embeddings generated for each group were then aggregated into 2D histograms (heatmaps) using a 300 $\times$ 300 binning scheme. This process yields discrete probability distributions over the embedding space. Seasonal changes were quantified using the Bhattacharyya coefficient, a measure of overlap between probability distributions.

To assess song stability and seasonal changes, we compared the following:

(1) Within-season stability: the average Bhattacharyya coefficient between same-season groups (e.g., breeding season 1 vs. breeding season 2 and non-breeding season 1 vs. non-breeding season 2).
(2) Between-season differentiation: the average Bhattacharyya coefficient between groups drawn from different seasons (e.g., breeding season 2 vs. non-breeding season 1).

Software packages

All neural network models, including TweetyBERT and the linear probes, were implemented and trained using PyTorch.[55] Spectrogram generation utilized Librosa[40] and SoundFile[41] libraries. Clustering was performed using a publicly available HDBSCAN package,[50] dimensionality reduction was achieved using UMAP,[56] and clustering performance was evaluated using V-measure from scikit-learn.[52] Numerical operations and array manipulations were performed using NumPy.[57] Figures and visualizations were created using Matplotlib.[58]

Computational resources

All model training and analyses were performed using PyTorch on a system equipped with an NVIDIA GeForce RTX 4090 GPU (24 GB VRAM), CUDA 12.3, driver v.545.23.08, and an AMD Ryzen threadripper pro 5955wx processor running Ubuntu 22.04.4 LTS with 128 GB of RAM. Approximately 2 TB of storage supported the intermediate computations required.

Dataset acquisition, collection, and annotation

This study utilized two datasets of adult male American Singer canaries (*Serinus canaria*): the TweetyNET dataset and a newly collected seasonal dataset. The TweetyNET dataset contained recordings and syllable-level annotations from three birds and has been described in detail previously.[5] To summarize this prior work, the syllable-level annotations were generated through a bootstrapped, semi-automatic approach: a small hand-labeled seed set was used to train the original TweetyNET model, which then automatically annotated the remaining data. Human experts subsequently proofread and corrected these automated annotations. These ground-truth labels provided the benchmark against which TweetyBERT's unsupervised clustering performance was evaluated.

The seasonal dataset comprised recordings from two birds across both breeding and non-breeding seasons. For this dataset, we only labeled recordings at the song level, marking which season each recording was collected from rather than providing syllable-level annotations. Recordings were made using an omnidirectional microphone (Audio-Technica AT803) positioned above each cage. The audio was routed through an M-Audio 8 pre-amplifier and captured using Sound Analysis Pro[11] software. Recordings were conducted at 44.1 kHz (single channel), generating WAV files.

For both datasets, song segments were isolated from all recordings using a supervised "song detector" (Figure S2), which eliminated calls, cage noise, and extended silences typically present in raw recordings.

Further details regarding the acquisition of data can be found in the supplemental information.

RESULTS

Emergence of structured representations in TweetyBERT embeddings

If birdsong syllables consisted of simple notes such as the sounds produced by piano keys, then dimensionality reduction methods (such as UMAP) applied directly to spectrogram images would clearly separate syllables into distinct clusters. However, canary syllables—and animal vocalizations more generally—require a consideration of the time course of acoustic elements to properly categorize vocalizations. This complexity is illustrated in Figure 3C, where UMAP dimensionality reduction was applied to individual spectrogram time bins (frequency vectors at single time points; 2.7 ms each) from three different canary songs. Here, each point corresponds to a single spectrogram time bin, colored according to ground-truth syllable labels from a previously described public dataset.[6] Black points are inter-syllabic silences. Different canary syllables share highly similar spectral features when analyzed at the single time-bin level (Figure 3C), causing substantial overlap between syllable classes in the embedding space.

In contrast, dimensionality reduction applied to TweetyBERT's internal latent representation reveals a structured embedding space in which the units of song—syllables—are distinctly separable from one another (Figure 3B). Each dimensionally reduced latent point still corresponds to 2.7 ms of birdsong but now enriched with temporal context from the surrounding sequence through the transformer's attention mechanism. In this representation, syllables correspond to elliptical trajectories in the 2D UMAP space. Points along an ellipse represent sequential temporal positions within a syllable's acoustic trajectory, and a full traversal corresponds to the complete utterance of that syllable. Remarkably, across hundreds of accumulated songs, utterances of the same syllable type consistently embed in the same latent-space location, forming these characteristic elliptical patterns—a consistency absent in the raw spectrogram embeddings where syllable instances scatter across overlapping regions. Repeated syllables within a phrase trace the same ellipse multiple times, demonstrating the model's ability to recognize syllable identity despite acoustic variations across renditions.

Acoustic interpretation of overlapping ellipses

We occasionally observed regions of intersection or proximity between ellipses representing different syllable classes. Spectrogram analysis reveals that these overlapping regions often result from genuine acoustic similarities rather than limitations in the model's representational capacity. For example, distinct syllable types sometimes share similar spectral properties during their onset, diverging only later in the syllable (Figure 3A). This partial acoustic overlap leads to intersections in the embedding space, reflecting real acoustic relationships.

Context-dependent representation of silences

TweetyBERT encodes silences differently depending on their context within the song. Inter-syllabic silences—brief pauses between syllables—are embedded within the elliptical trajectories of their corresponding syllable classes. In contrast, extended

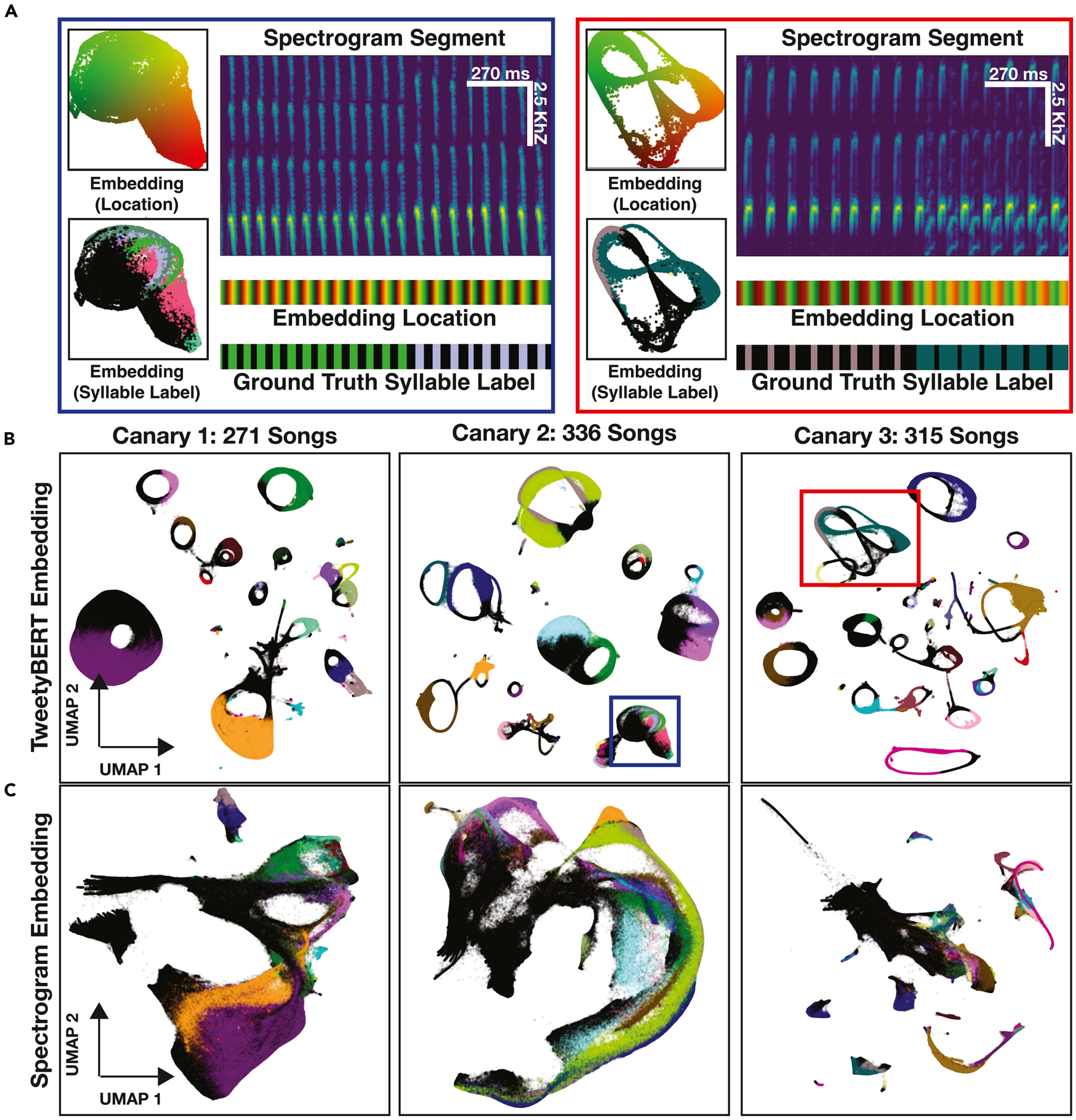

Figure 3. TweetyBERT and spectrogram UMAP embeddings

(A) Detailed analysis of syllables with more complex or overlapping latent-space representations.

(B) Learned latent representations from three different canaries colored by human-annotated syllable classes; 1 million time bins, colors denote syllable label.

(C) UMAP of individual frequency vectors (baseline comparison); 1 million time bins, colors denote syllable label.

silences found at song boundaries or between phrase transitions form a distinct, separate non-elliptical structure. Occasionally, this cluster also includes non-song elements, such as calls or background noise that were not fully excluded by the song detection step. This differentiation demonstrates the model's capacity to distinguish silences that form part of a repeated syllable from longer periods of non-vocalization or noise.

Clustering TweetyBERT's latent representation

TweetyBERT's embedding space demonstrates a structured organization suitable for efficient clustering (Figure 4). We used HDBSCAN due to its ability to automatically determine the number of clusters, adapt to variable cluster shapes, and handle noise effectively.[49] Its computational complexity of $O(n \log n)$ makes it suitable for analyzing large datasets (up to ~1 million time bins with 128 GB of RAM). While clustering could be performed on higher-dimensional UMAP representations or even the original latent space, we chose two dimensions for both theoretical and practical reasons. Clustering in high-dimensional spaces suffers from the curse of dimensionality, where points become increasingly isolated, requiring exponentially more data points to achieve successful clustering. Furthermore, varying the level of dimensionality reduction had a negligible effect on clustering performance, replicating findings from the literature,[5] making 2D projection sufficient for both visualization and clustering purposes (Figure S5).

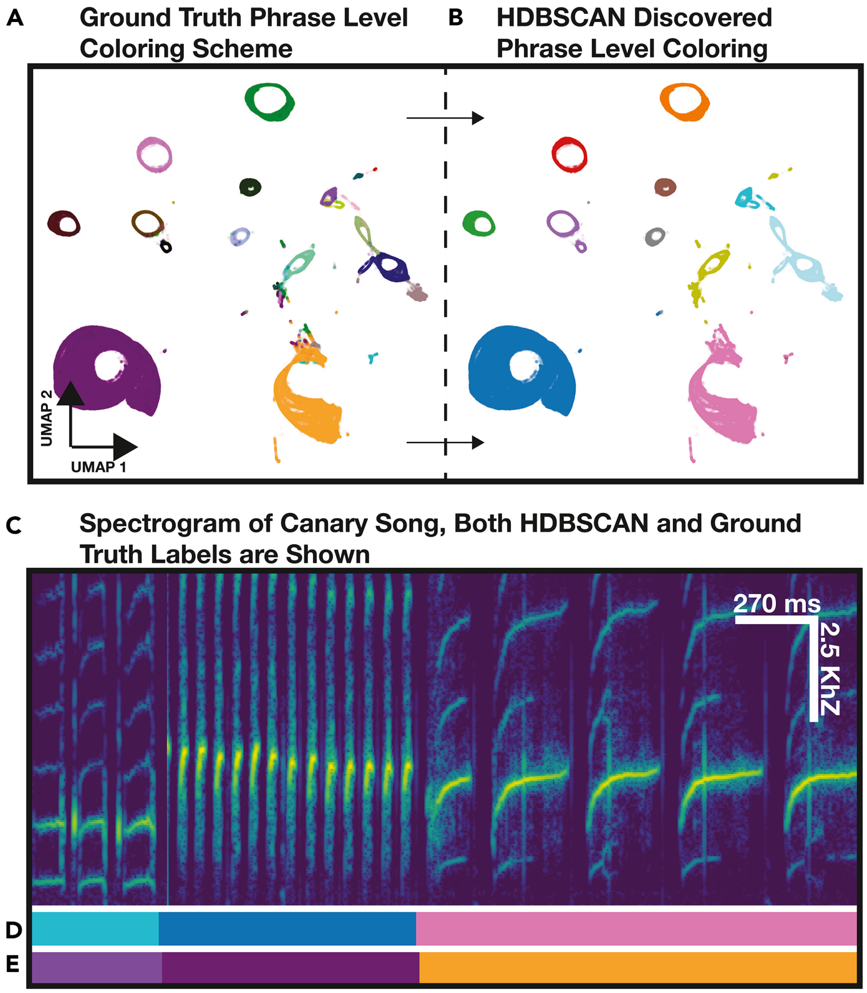

Figure 4. Machine-derived clusters and human-derived clusters are highly similar

(A) UMAP projection of the TweetyBERT latent representation colored by human ground-truth phrase labels.

(B) Same embeddings colored by phrase clusters identified via HDBSCAN.

(C) Spectrogram of canary song.

(D and E) HDBSCAN-derived labels (D) and ground-truth labels (E) illustrating agreement between unsupervised clustering and manual annotations.

Clustering evaluation

To quantitatively evaluate clustering performance, we applied the V-measure statistic,[51] a metric previously used in zebra finch and Bengalese finch vocalization studies.[4,59] V-measure is the harmonic mean of homogeneity (clusters contain one class) and completeness (each class forms a single cluster), ranging from 0 (no agreement) to 1 (perfect clustering). Clustering of TweetyBERT's latent representations for three canaries (partitioned into 12-fold due to UMAP memory constraints) resulted in a high V-measure score of 0.88 ± 0.02. These data were drawn from held-out audio data not seen during training. The V-measure score confirms strong alignment between automated clustering and ground-truth syllable classes, establishing a quantitative benchmark for unsupervised canary song clustering.

Reconciling labeling schemes

A minor technical issue arose because our available ground-truth data use different labels for syllables and the silences between syllables, even when those syllables are repeated in blocks of repetitions or "phrases" (Figure 1). For repeated syllables, HDBSCAN tends to cluster the inter-syllabic silences with the sound as a single elliptical trajectory. To accurately evaluate clustering performance relative to ground truth, we merged the syllable label with the inter-syllabic silence labels, as detailed in Figure S6.

Differences from human annotations

Although our clustering analysis showed strong overall agreement with human annotations, we identified specific common discrepancies. One difference was that HDBSCAN occasionally merged acoustically similar but distinct phrases into a single cluster—some of which may be difficult even for a human annotator to differentiate (Figure 3A). The comparison of the HDBSCAN cluster and ground-truth phrase labels is illustrated in Figures 4A and 4B.

We also observed fragmentation of single phrases into multiple clusters, particularly in complex syllables. In some of these instances, the fragmentation accurately identified meaningful sub-units of long-duration multi-part syllables (Figure 5A). This fragmentation is arguably a more meaningful description of the long multi-part syllables—and simply means that in these cases, the transformer discovers a different effective "convention" for parsing song than the convention followed by the human labeler. Whether errors or different conventions, all these departures from human labels will lower the V-measure score.

Limitations and potential improvements

While many discrepancies are valid alternative conventions for song parsing, a small number clearly constitute errors. For example, the insertion of spurious cluster labels—often lasting just a few time bins—represents misclassification errors (Figures 5A and 5B). Although these insertions are usually short in duration, they significantly impact downstream sequence analyses such as phrase durations, entropy measurements, and syntactic pattern analysis. Notably, these kinds of sequence structure disruptions are not readily detected by clustering analysis methods such as V-measure, which primarily assess cluster-to-class correspondence rather than temporal sequence integrity. These short errors can often be corrected through a

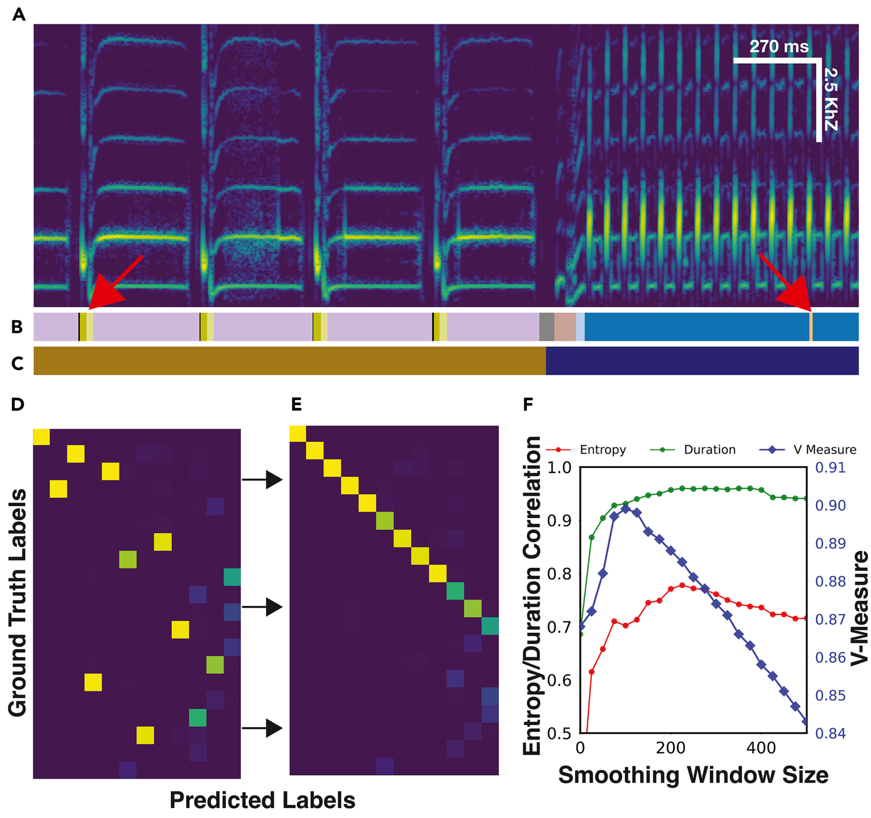

Figure 5. Comparing human and automated labels for sequence analysis

(A) Spectrogram of canary song with corresponding labels.

(B and C) HDBSCAN-generated clusters (B) and ground-truth human annotations (C). Red arrows indicate labeling discrepancies: syllable fragmentation (left) and spurious cluster insertions (right).

(D and E) Confusion matrices comparing predicted and ground-truth labels before (D) and after (E) alignment optimization. Misaligned or fragmented syllable predictions appear as off-diagonal elements, whereas unmatched machine-generated clusters occupy the lower rows of (E).

(F) Pearson correlations between HDBSCAN-derived and ground-truth labels for entropy, duration, and V-measure as a function of smoothing window size. Optimal smoothing aligns human and model annotations at maximal correlation.

post-processing step of temporally smoothing the syllable labels. This smoothing will be further discussed later.

Mapping machine to human labels

As mentioned earlier, automated (machine) cluster assignments occasionally contain fragmented classifications that distort syntactic structure, a type of error that is not reliably captured by V-measure. We devised a battery of evaluations to provide a more comprehensive picture of the performance of the model with respect to these kinds of errors, as well as the improvement of these metrics with the application of post-processing label smoothing.

For the first type of evaluation, we established a mapping between ground-truth and machine labels by constructing a co-occurrence matrix and then, using the Hungarian algorithm, created a linear mapping between the two label schemes.[60] This allowed us to compute the FER, enabling direct comparison of unsupervised clustering with supervised linear probe and fine-tuned performance of TweetyBERT, which is discussed in the next section. Second, we computed syntactic statistics (phrase duration and transition entropy) to verify that predicted cluster labels preserve the sequential structure statistics of ground-truth labels, which is essential for researchers seeking to use TweetyBERT to label canary songs.

Classification accuracy: FER

We introduced two variants of the FER to quantify two distinct types of error that arise from the linear mapping between machine and ground-truth labels: errors within mapped clusters and errors from unmapped clusters.

The total FER provides a comprehensive measure of overall classification accuracy. Using the "Rosetta Stone" mapping from the Hungarian algorithm, we first convert each discovered cluster label to its corresponding ground-truth label (if a mapping exists). We then compute the error rate by comparing these converted labels against the actual ground truth at every time bin. Crucially, any time bins assigned to unmapped clusters—those without a corresponding ground-truth label in the mapping—are automatically counted as errors. This directly penalizes under-clustering: when the model discovers fewer clusters than ground-truth labels, more clusters remain unmapped, resulting in a higher total FER.

The matched-only FER focuses specifically on the accuracy of mapped clusters. This metric considers only time bins whose predicted cluster has a valid mapping to a ground-truth class, measuring how well these mapped clusters align with their assigned ground-truth labels. The Hungarian algorithm produces a one-to-one mapping at the class level, but this does not guarantee frame-perfect alignment. For example, a cluster matched to phrase type A may still extend into regions labeled as phrase type B, creating frame-level errors at boundaries. The matched-only FER quantifies these boundary misalignments and within-cluster errors, providing insight into the temporal precision of successfully mapped clusters.

Syntactic structure: Phrase statistics

Beyond frame-level accuracy, we evaluated whether the model preserves the temporal organization and sequential patterns of birdsong by comparing higher-order statistical measures between human and machine labels. Specifically, we focused on phrase duration (the average length in time bins of each phrase type) and phrase entropy (the breadth of transition probabilities between states). We chose these metrics because they are established measures in the birdsong literature for characterizing song syntax.[9,12] Phrase duration reveals whether the model

correctly parses phrase boundaries—oversized smoothing windows artificially join distinct phrases, reducing correlation with ground-truth durations. Transition entropy tests whether the model captures sequential dependencies between phrases—undersized smoothing windows introduce spurious transitions that fragment natural sequences and destroy syntactic patterns. We compute the correlation between phrase duration and phrase entropy derived from both human and machine annotations for each matched phrase type in the dataset.

Effect of smoothing window size

To both preserve the syntactic structure of the song, as well as mitigate label fragmentation, we explore the application of temporal smoothing, a procedure that moves a window across the sequence and, for each position, reassigns the center time bin based on the most frequently occurring state within its surrounding window. We hypothesize that an optimal smoothing window size exists that balances removing spurious brief misclassifications (which artificially fragment phrases and distort syntax) against preserving genuine phrase boundaries (which define natural song structure).

We varied the temporal smoothing window from 0 to 500 time bins in increments of 25 time bins. Smaller windows (0–50 time bins) yielded more fragmented classifications, higher FER scores (matched-only FERs $\geq$ 6.76%, total FERs $\geq$ 15.12%), and lower entropy correlations ($r \leq 0.658$), despite achieving high V-measure scores (up to 0.882). Moderate smoothing windows (150–225 time bins) substantially improved overall performance, resulting in strong phrase duration correlations ($r \geq 0.947$), significantly improved entropy correlations ($r \geq 0.745$), and lower error rates (matched-only FERs $\leq$ 4.99%, total FERs $\leq$ 14.52%). Optimal performance was achieved at a 200-time-bin window ($\sim$540 ms), achieving the lowest matched-only FER (4.34%), a low total FER (13.97%), and the highest entropy correlation (r = 0.771), albeit with a slight decrease in V-measure (0.888 vs. 0.893 at 150 bins) (Table S2; Figure S7).

Recommendations and practical implications

For canary song, we recommend a default smoothing window of approximately 200 time bins ($\sim$540 ms), as this window size balances cluster fragmentation, classification accuracy, and entropy correlations effectively, and this duration corresponds roughly to the duration of most canary phrases.[9] However, the ideal window size ultimately depends on analytical goals, such as correct classification of phrases or preserving the syntactic structure of the song. Practically, smoothing windows between 150 and 225 bins offer robust compromise solutions, effectively capturing essential syntactic structure while minimizing labeling errors.

A linear probe alternative to clustering

The previous analyses of TweetyBERT's latent representations relied on UMAP dimensionality reduction combined with clustering. While useful for visualization and clustering, UMAP is highly nonlinear, limiting the interpretability of the benchmarks described in the previous section. Additionally, squeezing high-dimensional spaces into lower dimensions inevitably causes information loss, and the UMAP and HDBSCAN clustering results are specific to hyperparameter choices. To complement the UMAP analysis, we turned to linear probes, a widely used method for evaluating neural network embeddings without reliance on nonlinear dimensionality reduction techniques.

Linear probes are simple linear classifiers—a single matrix multiplication—applied directly to a network's internal activations that are used to predict class labels.[61] These classifiers cannot create new representations; they can only rotate or scale existing ones. Such methods have demonstrated that self-supervised transformers encode sophisticated semantic and syntactic structures without explicit supervision.[62–64] High performance of a linear probe would indicate that the underlying latent spaces are meaningfully structured.

Here, we use linear probes to evaluate whether TweetyBERT's self-supervised training produces emergent phrase-level representations of canary song. To apply the linear probe to the model, we simply train a linear classifier to reproduce human phrase labels, using the latent-space vectors produced by TweetyBERT.

Experimental conditions for the linear probe

We examined 4 different models, trained to reproduce the human labels. To maintain consistency with earlier analyses in this study, we used the latent representations from the multi-head self-attention layer of the 3rd transformer block as the basis for linear probe classification—the same layer used for clustering and UMAP visualization throughout.

(1) TweetyBERT fine-tuning: the first test is not a linear probe but rather a full fine-tuning of all parameters of TweetyBERT to reproduce the human labels. This provided the best FER that can be achieved with the TweetyBERT architecture if full supervised training is allowed.
(2) TweetyBERT linear probe: the probe was applied to the self-supervised (trained) TweetyBERT model. All parameters other than the linear projection were frozen after self-supervised pretraining.
(3) Untrained TweetyBERT linear probe: the probe was applied to the outputs of the untrained (randomly initialized) TweetyBERT model. All parameters other than the linear projection were frozen. This condition assesses the inherent inductive biases of the untrained architecture.
(4) Spectrogram linear probe: the linear probe was applied directly to the raw spectrogram features. Specifically, the linear probe is trained to assign a syllable label to each individual 196D spectral vector (single time bin) independently, without access to temporal context from neighboring time bins. This approach confirms that phrases are not linearly separable based on their instantaneous spectral features alone, as phrase identity inherently depends on temporal patterns rather than isolated spectral snapshots. The temporal resolution of these spectral vectors matches the temporal resolution of the TweetyBERT model (2.7 ms), making it a useful baseline comparison.

Linear probe results

To assess the quality of learned representations, we computed the FER to measure the percentage of time bins where predicted

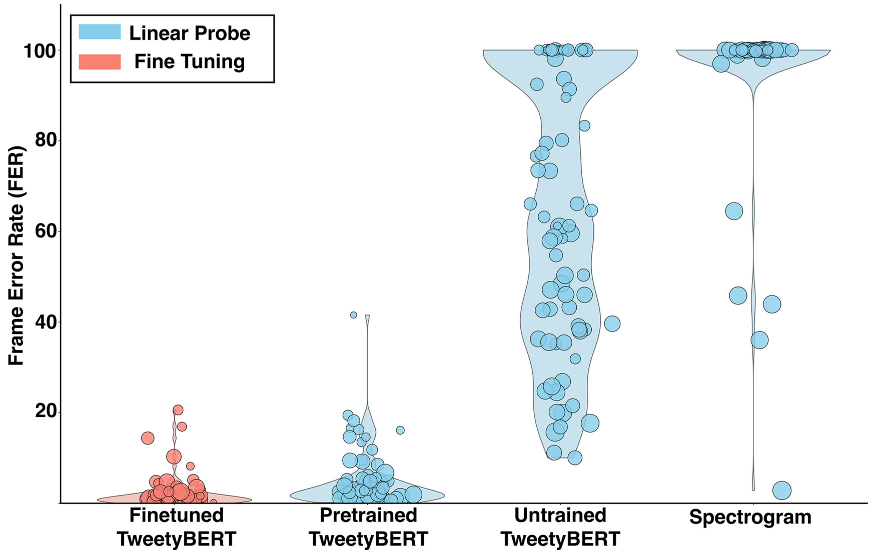

Figure 6. Evaluating TweetyBERT embeddings using linear probes

Frame error rates (FERs) comparing fine-tuned TweetyBERT to linear probes on embeddings from pretrained TweetyBERT models, untrained TweetyBERT models, and spectrograms. Embeddings from pretrained TweetyBERT achieve near-fine-tuned accuracy, substantially surpassing those from untrained TweetyBERT or raw spectrogram features.

phrase labels disagree with ground-truth annotations. FER scores were derived from a held-out dataset not used during pretraining or linear probe training. On these unseen data, the linear probe applied to pretrained TweetyBERT achieved an average total FER of 2.5% across birds, though performance varied across phrase types (standard deviation between individual phrase FERs: 6.3%) (Figure 6). The fully supervised fine-tuned model performed better overall (FER: 1.3%), with more consistent class performance (standard deviation: 3.5%), indicating that self-supervised learning alone produces near-optimal phrase-level representations. In contrast, the unsupervised clustering from earlier achieved a 13.97% total FER at optimal smoothing, indicating room for further optimization. As expected, the untrained TweetyBERT performed worse (FER: 45.1%, standard deviation: 30.0%) yet surpassed the raw spectrogram baseline (FER: 82.6%, standard deviation: 15.8%). Since both the untrained model and the raw spectrogram classifier used identical 196D input spaces, the considerable performance advantage (~38%) of the untrained transformer indicates that its architecture inherently generates latent variables that mix information from distinct time points, providing a boost in performance. This observation aligns with studies suggesting that transformers have intrinsic inductive biases beneficial for temporally structured tasks[65] and that, more broadly, linear readouts over random features can be highly effective,[66,67] and in this sense, the untrained transformer functions analogously to a reservoir computer or liquid state machine.[68,69]

Implications for architecture selection

TweetyBERT's randomly initialized architecture provides a meaningful boost in classification, and this suggests that linear probes of randomly initialized networks could be used to select hyperparameters or architectures with favorable inductive biases before large-scale training. This approach demonstrates that transformers possess inherent architectural advantages for birdsong analysis, specifically their ability to automatically integrate temporal information across multiple timescales. Unlike previous methods that require explicit windowing strategies or syllable segmentation to capture phrase-level patterns, the transformer architecture naturally aggregates context from surrounding time bins through its attention mechanism, eliminating the need for manual temporal feature engineering. The self-supervised pretraining further refines these initial representations, resulting in phrase-level embeddings that closely approximate supervised fine-tuned performance.

TweetyBERT reveals seasonal vocal plasticity in canaries

Canaries are seasonal songbirds known to exhibit an annual cycle of song relearning. The underlying mechanisms of neural plasticity include large-scale neural replacement that provides canary brains with newborn neurons capable of song relearning each fall.[35,36] To investigate how TweetyBERT captures these seasonal differences, we trained a new model combining spring and fall song data from two birds. From this model, we generated embeddings comprising approximately 1 million time bins per bird, divided evenly into four temporally distinct subsets (~250,000 bins each): two subsets from the breeding (spring) and two from the non-breeding (fall) seasons. All spring songs were color coded purple and fall songs green (Figure 7). We note that the spring songs do not appear as well organized as prior UMAP representations of spring songs generated by TweetyBERT. This is likely because the UMAP embedding combined spring and fall recordings, which have different acoustic characteristics.

To further quantify embedding similarity, we constructed 300 × 300 binned heatmaps representing point densities in UMAP space and quantified the overlap of these densities using the Bhattacharyya coefficient. Within-season comparisons revealed high stability of these UMAP density plots for both canaries (canary 1: breeding = 0.938 and non-breeding = 0.841; canary 2: breeding = 0.926 and non-breeding = 0.930). However, between-season analyses showed moderate overlap for canary 1 (0.477) and lower overlap for canary 2 (0.119), highlighting more substantial seasonal vocal reorganization in canary 2.

These observations align with prior reports on seasonal vocal plasticity in canaries. The consistently high within-season similarity scores indicate stable song that has been termed "crystalized song" by researchers, whereas the lower between-season similarity indicates significant repertoire restructuring in the transition between breeding season and fall, a pattern visually

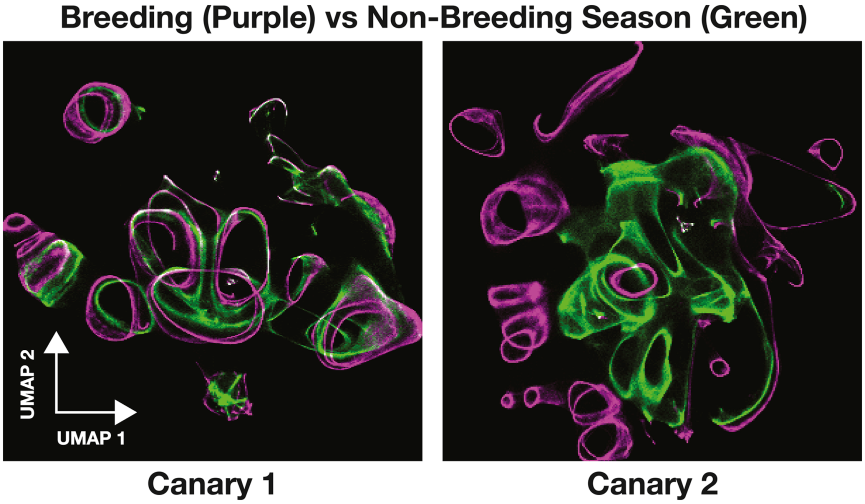

Figure 7. TweetyBERT embeddings vary by season of the year

UMAP embeddings of breeding season song (purple) and fall season song (green) of two canaries. Regions of overlap appear in white.

evident in the UMAP embedding of canary 1's vocalization. These observations in a small number of birds suggest that TweetyBERT embeddings may provide a useful latent space for a variety of song analysis tasks. Future studies will be needed to compare the performance of TweetyBERT relative to other unsupervised representations of song for qualitative analysis of latent spaces.

DISCUSSION

Limitations

Several practical challenges remain for broadly deploying self-supervised models such as TweetyBERT in animal communication studies. Foremost, a separate song detector network is likely essential to isolate songs from cage noise or other background sounds prior to training, as embeddings are known to perform better on downstream tasks when isolated from surrounding extraneous audio.[70] We have not examined whether TweetyBERT can be trained to parse song if cage noise or other environmental noises contaminate the recordings. If the model uses its representational capacity to predict these non-song acoustic elements, its performance on song will likely be reduced. In contexts where robust song detectors do not exist—particularly for niche or understudied species—developing custom detectors introduces additional overhead.

Although TweetyBERT effectively matches human labels for most syllables, some discrepancies persist between automated clusters and human-annotated labels. These differences in most cases reflect a difference of convention with human practice—such as splitting long multi-part syllables into distinct clusters or combining clusters that are not reliably separated. In some cases, syllables may be assigned to the wrong cluster or labeled as noise if they do not reliably fit into a clear phrase type, such as transition syllables. When this occurs, fuzzy clustering or membership probabilities from HDBSCAN might be employed to better handle these ambiguous cases, allowing syllables to belong partially to multiple classes simultaneously. Similarly, HDBSCAN failed to separate some partially overlapping acoustic trajectories. A history-dependent clustering approach, such as autoregressive hidden Markov models (AR-HMMs), may improve cluster separation by incorporating sequential dependencies.[71] In general, the selection of a specific clustering and evaluation algorithm—in our case, HDBSCAN and V-measure—is completely context dependent[72] and thus must be applied carefully.

Computational limitations present another barrier. UMAP dimensionality reduction becomes computationally prohibitive when processing large datasets comprising millions of data points, restricting scalability and reproducibility. While these limitations do not invalidate insights derived from smaller-scale analyses, they hinder the broader deployment of TweetyBERT for large-scale data analysis. Approaches to mitigate this limitation of UMAP include the development of decoders trained to predict the cluster labels generated by the TweetyBERT model so that the UMAP step can be avoided in large-scale inference tasks.

Finally, we focused this study solely on American Singer canaries and did not conduct broader comparisons with other unsupervised methods or apply TweetyBERT to non-canary datasets. Adaptation of TweetyBERT to different singers may require further work in tuning the architecture and hyperparameters.

Future directions

Pixelwise reconstruction of missing spectrogram patches is fragile in that small variations in the spectral or temporal structure of predicted spectrogram fragments can lead to significant variations in the reconstruction score. Similar limitations in image and video tasks motivated the recent development of latent-space predictive architectures such as joint-embedding predictive architectures (JEPAs).[73,74] Improving the objective function that guides self-supervised learning could yield more sample-efficient learning, which would be a win, as bioacoustics is a domain with relatively few high-quality data.[42] These more advanced models may be necessary to achieve robust performance with more complex vocalizations, such as juvenile song, or more complex acoustic environments, such as environmental recordings in the wild.

Future work may explore models incorporating internal dimensionality reduction, eliminating reliance on external methods such as UMAP, as demonstrated successfully in neural activity embeddings.[75] Furthermore, extending transformer context windows beyond the 2.7 s used here could improve performance by capturing longer-range dependencies in vocal sequences.[9]

Systematic hyperparameter tuning can be a challenge in training large-scale audio models. Our current selection of hyperparameters (e.g., context length, masking strategies, or number and size of transformer layers) was guided by current best

practices of parameter count to data ratios,[76] but we did not explore the model parameter space in any meaningful way. Still, current evaluation metrics depend on ground-truth human annotations, which may not always be available or unbiased. If machine learning provides a better parsing of song than humans do, how can we recognize this? Future measures of model representations could investigate whether machine-generated labels are more predictive of song syntax or spectral features than ground-truth annotations.

Our findings highlight the potential for self-supervised models such as TweetyBERT to enhance research in animal communication. While we have focused on models applied to single individuals singing alone, a range of groups are working on methods to capture social conversations.[77,78] Latent-space representations of vocal communication in social contexts could reveal how the structure of the vocalizations depends on the identity of the conversational partner or what that partner said. Analysis of social conversations in songbirds and other vocal communicators, such as parrots or dolphins, has already begun to reveal previously hidden meanings in vocal behavior,[79,80] with recent unsupervised clustering and latent-space modeling of sperm whale communication providing clear examples of this emerging science.[81–84]

Conclusions

TweetyBERT is a transformer model that learns the structure of birdsong in a self-supervised process, independent of any human input. With minimal preprocessing of sound, the model is trained to simply fill in gaps—masked data in raw spectrograms. In the process of solving this "fill-in-the-blank" task, the model develops an internal representation of song that corresponds to distinct syllables—the biophysical units of song production. Automated clustering of the model's song representations yields syllable clusters that correspond well with human annotations of canary song.

This emergence of a syllabic representation of song is a finding similar to the emergence of phonemic representations when self-supervised models are trained on raw speech.[33] While the model draws inspiration from these prior human speech models, TweetyBERT is designed and trained from scratch for birdsong—the temporal resolution of the model is an order of magnitude faster than human speech models, allowing for the fine-grained representation of birdsong.

In addition to providing automated clustering of song syllables, the latent space generated by the model can be used for the analysis of song variability, as suggested in our comparison of canary songs in the transition from spring breeding songs to fall plastic songs. Powerful new latent-space representations of song may provide new windows into the song learning process—a particularly challenging subject of study since juvenile songbirds produce highly variable songs, and prior advances in sound analysis for song have often uncovered new principles of that vocal learning process.

Finally, since the model involves minimal preprocessing of sound, we anticipate that similar architectures will be applicable to a wide range of species. When applied to field recordings or passive acoustic monitoring stations, models such as TweetyBERT may soon yield information about individual identities of songbirds, species abundance, or the impact of human infrastructure on animal lives as seen through the lens of vocal communication.

RESOURCE AVAILABILITY

Lead contact

For further inquiry about the data, results, and methods, contact Timothy J. Gardner (timg@uoregon.edu).

Materials availability

This study did not generate new unique reagents.

Data and code availability

The steps to replicate findings, figures, and numerical results are available at Zenodo (https://doi.org/10.5281/zenodo.15391040).[85] The TweetyNET dataset used for model training and ground-truth labels is publicly available via Dryad (https://doi.org/10.5061/dryad.xgxd254f4).[86]

ACKNOWLEDGMENTS

We would like to thank Diana Ostojich and Ananya Kapoor for providing edits and feedback and Ellen Sova for supporting the work on song detection. This work was funded by NIH R01NS118424.

AUTHOR CONTRIBUTIONS

T.J.G. and G.V. conceived the study, designed the algorithm, and co-wrote the manuscript. G.V. wrote all the code. M.A.B. and M.R.H.-V. conducted bird care, collected seasonality song recordings, and tested the algorithm. M.R.H.-V. assisted with seasonality data analysis and contributed to song detector creation. M.A.B. maintained animal protocols and designed and built the experimental cages.

DECLARATION OF INTERESTS

The authors declare no competing interests.

DECLARATION OF GENERATIVE AI AND AI-ASSISTED TECHNOLOGIES IN THE WRITING PROCESS

The authors used ChatGPT and Claude solely to rephrase, simplify, and improve the manuscript's prose and grammar. All suggestions were reviewed and approved by the authors prior to submission.

SUPPLEMENTAL INFORMATION

Supplemental information can be found online at https://doi.org/10.1016/j.patter.2025.101491.

Received: June 17, 2025
Revised: October 15, 2025
Accepted: December 30, 2025
Published: March 3, 2026

REFERENCES

1. Rutz, C., Bronstein, M., Raskin, A., Vernes, S.C., Zacarian, K., and Blasi, D.E. (2023). Using machine learning to decode animal communication. Science *381*, 152–155. https://doi.org/10.1126/science.adg7314.
2. Kahl, S., Wood, C.M., Eibl, M., and Klinck, H. (2021). BirdNET: A deep learning solution for avian diversity monitoring. Ecol. Inform. *61*, 101236. https://doi.org/10.1016/j.ecoinf.2021.101236.
3. Gu, N., Lee, K., Basha, M., Kumar Ram, S., You, G., and Hahnloser, R.H.R. (2024). Positive transfer of the whisper speech transformer to human and animal voice activity detection. In ICASSP 2024 - 2024 IEEE International Conference on Acoustics, Speech and Signal Processing (ICASSP) (IEEE), pp. 7505–7509. https://doi.org/10.1109/icassp48485.2024.10447620.

CellPress
OPEN ACCESS

4. Sainburg, T., Thielk, M., and Gentner, T.Q. (2020). Finding, visualizing, and quantifying latent structure across diverse animal vocal repertoires. PLoS Comput. Biol. *16*, e1008228. https://doi.org/10.1371/journal.pcbi.1008228.
5. Best, P., Paris, S., Glotin, H., and Marxer, R. (2023). Deep audio embeddings for vocalisation clustering. PLoS One *18*, e0283396. https://doi.org/10.1371/journal.pone.0283396.
6. Cohen, Y., Nicholson, D.A., Sanchioni, A., Mallaber, E.K., Skidanova, V., and Gardner, T.J. (2022). Automated annotation of birdsong with a neural network that segments spectrograms. eLife *11*, e63853. https://doi.org/10.7554/eLife.63853.
7. Briefer, E.F., Sypherd, C.C.-R., Linhart, P., Leliveld, L.M.C., Padilla de la Torre, M., Read, E.R., Guérin, C., Deiss, V., Monestier, C., Rasmussen, J.H., et al. (2022). Classification of pig calls produced from birth to slaughter according to their emotional valence and context of production. Sci. Rep. *12*, 3409. https://doi.org/10.1038/s41598-022-07174-8.
8. Cohen, Y., Shen, J., Semu, D., Leman, D.P., Liberti, W.A., 3rd, Perkins, L.N., Liberti, D.C., Kotton, D.N., and Gardner, T.J. (2020). Hidden neural states underlie canary song syntax. Nature *582*, 539–544. https://doi.org/10.1038/s41586-020-2397-3.
9. Markowitz, J.E., Ivie, E., Kligler, L., and Gardner, T.J. (2013). Long-range order in canary song. PLoS Comput. Biol. *9*, e1003052. https://doi.org/10.1371/journal.pcbi.1003052.
10. Kobayashi, K., Uno, H., and Okanoya, K. (2001). Partial lesions in the anterior forebrain pathway affect song production in adult Bengalese finches. Neuroreport *12*, 353–358. https://doi.org/10.1097/00001756-200102120-00034.
11. Veit, L., Tian, L.Y., Monroy Hernandez, C.J., and Brainard, M.S. (2021). Songbirds can learn flexible contextual control over syllable sequencing. eLife *10*, e61610. https://doi.org/10.7554/eLife.61610.
12. Koparkar, A., Warren, T.L., Charlesworth, J.D., Shin, S., Brainard, M.S., and Veit, L. (2024). Lesions in a songbird vocal circuit increase variability in song syntax. eLife *13*, RP93272. https://doi.org/10.7554/eLife.93272.
13. Tachibana, R.O., Oosugi, N., and Okanoya, K. (2014). Semi-automatic classification of birdsong elements using a linear support vector machine. PLoS One *9*, e92584. https://doi.org/10.1371/journal.pone.0092584.
14. Koumura, T., and Okanoya, K. (2016). Automatic recognition of element classes and boundaries in the birdsong with variable sequences. PLoS One *11*, e0159188. https://doi.org/10.1371/journal.pone.0159188.
15. Daou, A., Johnson, F., Wu, W., and Bertram, R. (2012). A computational tool for automated large-scale analysis and measurement of bird-song syntax. J. Neurosci. Methods *210*, 147–160. https://doi.org/10.1016/j.jneumeth.2012.07.020.
16. Steinfath, E., Palacios-Muñoz, A., Rottschäfer, J.R., Yuezak, D., and Clemens, J. (2021). Fast and accurate annotation of acoustic signals with deep neural networks. eLife *10*, e68837. https://doi.org/10.7554/eLife.68837.
17. Singh Alvarado, J., Goffinet, J., Michael, V., Liberti, W., 3rd, Hatfield, J., Gardner, T., Pearson, J., and Mooney, R. (2021). Neural dynamics underlying birdsong practice and performance. Nature *599*, 635–639. https://doi.org/10.1038/s41586-021-04004-1.
18. Goffinet, J., Brudner, S., Mooney, R., and Pearson, J. (2021). Low-dimensional learned feature spaces quantify individual and group differences in vocal repertoires. eLife *10*, e67855. https://doi.org/10.7554/eLife.67855.
19. Tchernichovski, O., Nottebohm, F., Ho, C.E., Pesaran, B., and Mitra, P.P. (2000). A procedure for an automated measurement of song similarity. Anim. Behav. *59*, 1167–1176. https://doi.org/10.1006/anbe.1999.1416.
20. Vaswani, A., Shazeer, N., Parmar, N., Uszkoreit, J., Jones, L., Gomez, A.N., Kaiser, Ł., and Polosukhin, I. (2017). Attention is all you need. Adv. Neural Inf. Process. Syst. *30*, 5998–6008.
21. Devlin, J., Chang, M.-W., Lee, K., and Toutanova, K. (2019). BERT: Pre-training of Deep Bidirectional Transformers for Language Understanding. In Proceedings of the 2019 Conference of the North American Chapter of the Association for Computational Linguistics: Human Language Technologies, Volume 1 (Long and Short Papers) (Association for Computational Linguistics), pp. 4171–4186. https://doi.org/10.18653/v1/N19-1423.
22. Touvron, H., Lavril, T., Izacard, G., Martinet, X., Lachaux, M.-A., Lacroix, T., Rozière, B., Goyal, N., Hambro, E., Azhar, F., et al. (2023). Llama: Open and efficient foundation language models. Preprint at arXiv. https://doi.org/10.48550/arXiv.2302.13971.
23. Radford, A., Narasimhan, K., Salimans, T., and Sutskever, I. (2018). Improving language understanding by generative pre-training. OpenAI. https://cdn.openai.com/research-covers/language-unsupervised/language_understanding_paper.pdf.
24. Hsu, W.-N., Bolte, B., Tsai, Y.-H.H., Lakhotia, K., Salakhutdinov, R., and Mohamed, A. (2021). Hubert: Self-supervised speech representation learning by masked prediction of hidden units. IEEE/ACM Trans. Audio Speech Lang. Process. *29*, 3451–3460. https://doi.org/10.1109/TASLP.2021.3122291.
25. Dosovitskiy, A., Beyer, L., Kolesnikov, A., Weissenborn, D., Zhai, X., Unterthiner, T., Dehghani, M., Minderer, M., Heigold, G., Gelly, S., et al. (2021). An image is worth 16x16 words: Transformers for image recognition at scale. In International Conference on Learning Representations (ICLR) https://openreview.net/forum?id=YicbFdNTTy.
26. Radford, A., Kim, J.W., Hallacy, C., Ramesh, A., Goh, G., Agarwal, S., Sastry, G., Askell, A., Mishkin, P., Clark, J., et al. (2021). Learning transferable visual models from natural language supervision. In Proceedings of the 38th International Conference on Machine Learning, M. Meila and T. Zhang, eds. (PMLR), pp. 8748–8763.
27. Hagiwara, M. (2023). Aves: Animal vocalization encoder based on self-supervision. In ICASSP 2023-2023 IEEE International Conference on Acoustics, Speech and Signal Processing (ICASSP) (IEEE), pp. 1–5. https://doi.org/10.1109/ICASSP49357.2023.10095642.
28. Deng, X., Wan, T., Xu, K., Gao, T., Qiao, P., Feng, D., and Dou, Y. (2025). Scaling Bioacoustic Signal Pre-training with Million Samples Via Mask-Modeling. In ICASSP 2025-2025 IEEE International Conference on Acoustics, Speech and Signal Processing (ICASSP) (IEEE), pp. 1–5. https://doi.org/10.1109/ICASSP49660.2025.10890750.
29. Sarkar, E., and Magimai-Doss, M. (2023). Can self-supervised neural representations pre-trained on human speech distinguish animal callers? In Interspeech 2023 (ISCA). https://doi.org/10.21437/interspeech.2023-1968.
30. Cauzinille, J., Favre, B., Marxer, R., Clink, D., Ahmad, A.H., and Rey, A. (2024). Investigating self-supervised speech models' ability to classify animal vocalizations: The case of gibbon's vocal signatures. In Interspeech 2024 (ISCA). https://doi.org/10.21437/interspeech.2024-1096.
31. Ghani, B., Denton, T., Kahl, S., and Klinck, H. (2023). Global birdsong embeddings enable superior transfer learning for bioacoustic classification. Sci. Rep. *13*, 22876. https://doi.org/10.1038/s41598-023-49989-z.
32. Kloots, M. de H., and Knörnschild, M. (2024). Exploring bat song syllable representations in self-supervised audio encoders. Preprint at arXiv. https://doi.org/10.48550/arXiv.2409.12634.
33. Baevski, A., Zhou, Y., Mohamed, A., and Auli, M. (2020). wav2vec 2.0: A framework for self-supervised learning of speech representations. Adv. Neural Inf. Process. Syst. *33*, 12449–12460. https://doi.org/10.5555/3495724.3496768.
34. Gardner, T., Cecchi, G., Magnasco, M., Laje, R., and Mindlin, G.B. (2001). Simple motor gestures for birdsongs. Phys. Rev. Lett. *87*, 208101. https://doi.org/10.1103/physrevlett.87.208101.
35. Nottebohm, F., Nottebohm, M.E., and Crane, L. (1986). Developmental and seasonal changes in canary song and their relation to changes in the anatomy of song-control nuclei. Behav. Neural. Biol. *46*, 445–471. https://doi.org/10.1016/s0163-1047(86)90485-1.
36. Voigt, C., and Leitner, S. (2008). Seasonality in song behaviour revisited: seasonal and annual variants and invariants in the song of the domesticated canary (Serinus canaria). Horm. Behav. *54*, 373–378. https://doi.org/10.1016/j.yhbeh.2008.05.001.

37. Liu, A.T., Li, S.-W., and Lee, H.-Y. (2021). TERA: Self-Supervised Learning of Transformer Encoder Representation for Speech. IEEE/ACM Trans. Audio Speech Lang. Process. *29*, 2351–2366. https://doi.org/10.1109/TASLP.2021.3095662.

38. Rauch, L., Heinrich, R., Moummad, I., Joly, A., Sick, B., and Scholz, C. (2025). Can masked autoencoders also listen to birds?. Preprint at arXiv. https://doi.org/10.48550/arXiv.2504.12880.

39. Amy, M., Salvin, P., Naguib, M., and Leboucher, G. (2015). Female signalling to male song in the domestic canary, Serinus canaria. R. Soc. Open Sci. *2*, 140196. https://doi.org/10.1098/rsos.140196.

40. McFee, B., McVicar, M., Balke, S., Thomé, C., Raffel, C., Nieto, O., Battenberg, E., Ellis, D., Yamamoto, R., Moore, J., et al. (2018). Librosa. Zenodo. https://doi.org/10.5281/zenodo.1174893.

41. Bechtold, B. (2025). SoundFile: An audio library based on libsndfile, CFFI and NumPy (Version 0.13.1). GitHub. https://github.com/bastibe/python-soundfile.

42. van Merriënboer, B., Dumoulin, V., Hamer, J., Harrell, L., Burns, A., and Denton, T. (2025). Perch 2.0: The Bittern Lesson for Bioacoustics. Preprint at arXiv. https://doi.org/10.48550/arXiv.2508.04665.

43. Gulati, A., Qin, J., Chiu, C.-C., Parmar, N., Zhang, Y., Yu, J., Han, W., Wang, S., Zhang, Z., Wu, Y., et al. (2020). Conformer: Convolution-augmented Transformer for Speech Recognition. In Interspeech 2020 (ISCA), pp. 5036–5040. https://doi.org/10.21437/interspeech.2020-3015.

44. Shaw, P., Uszkoreit, J., and Vaswani, A. (2018). Self-Attention with Relative Position Representations. In Proceedings of the 2018 Conference of the North American Chapter of the Association for Computational Linguistics: Human Language Technologies, Volume 2 (Short Papers) (Association for Computational Linguistics), pp. 464–468. https://doi.org/10.18653/v1/N18-2074.

45. Hendrycks, D., and Gimpel, K. (2016). Gaussian Error Linear Units (GELUs). Preprint at arXiv. https://doi.org/10.48550/arXiv.1606.08415.

46. Xiong, R., Yang, Y., He, D., Zheng, K., Zheng, S., Xing, C., Zhang, H., Lan, Y., Wang, L., and Liu, T.-Y. (2020). On layer normalization in the Transformer architecture. In Proceedings of the 37th International Conference on Machine Learning, *119*, H. Daumé, III and A. Singh, eds. (PMLR), pp. 10524–10533.

47. Lasseck, M. (2018). Audio-based Bird Species Identification with Deep Convolutional Neural Networks. In Working Notes of CLEF 2018 - Conference and Labs of the Evaluation, pp. 1–11.

48. Kingma, D.P., and Ba, J. (2015). Adam: A Method for Stochastic Optimization. In International Conference on Learning Representations (ICLR).

49. Micikevicius, P., Narang, S., Alben, J., Diamos, G., Elsen, E., Garcia, D., Ginsburg, B., Houston, M., Kuchaiev, O., Venkatesh, G., et al. (2018). Mixed precision training. In International Conference on Learning Representations (ICLR).

50. McInnes, L., Healy, J., and Astels, S. (2017). hdbscan: Hierarchical density based clustering. J. Open Source Softw. *2*, 205. https://doi.org/10.21105/joss.00205.

51. Rosenberg, A., and Hirschberg, J. (2007). V-measure: A Conditional Entropy-Based External Cluster Evaluation Measure. In Proceedings of the 2007 Joint Conference on Empirical Methods in Natural Language Processing and Computational Natural Language Learning (EMNLP-CoNLL) (Association for Computational Linguistics), pp. 410–420.

52. Pedregosa, F., Varoquaux, G., Gramfort, A., Michel, V., Thirion, B., Grisel, O., Blondel, M., Prettenhofer, P., Weiss, R., Dubourg, V., et al. (2011). Scikit-learn: Machine Learning in Python. J. Mach. Learn. Res. *12*, 2825–2830.

53. Munkres, J. (1957). Algorithms for the Assignment and Transportation Problems. J. Soc. Ind. Appl. Math. *5*, 32–38. https://doi.org/10.1137/0105003.

54. He, K., Zhang, X., Ren, S., and Sun, J. (2015). Delving Deep into Rectifiers: Surpassing Human-Level Performance on ImageNet Classification. In Proceedings of the IEEE International Conference on Computer Vision (ICCV) (IEEE), pp. 1026–1034. https://doi.org/10.1109/ICCV.2015.123.

55. Paszke, A., Gross, S., Massa, F., Lerer, A., Bradbury, J., Chanan, G., Killeen, T., Lin, Z., Gimelshein, N., Antiga, L., et al. (2019). PyTorch: An Imperative Style, High-Performance Deep Learning Library. Adv. Neural Inf. Process. Syst. *32*, 8024–8035.

56. McInnes, L., Healy, J., Saul, N., and Großberger, L. (2018). UMAP: Uniform Manifold Approximation and Projection. J. Open Source Softw. *3*, 861. https://doi.org/10.21105/joss.00861.

57. Harris, C.R., Millman, K.J., van der Walt, S.J., Gommers, R., Virtanen, P., Cournapeau, D., Wieser, E., Taylor, J., Berg, S., Smith, N.J., et al. (2020). Array programming with NumPy. Nature *585*, 357–362. https://doi.org/10.1038/s41586-020-2649-2.

58. Hunter, J.D. (2007). Matplotlib: A 2D Graphics Environment. Comput. Sci. Eng. *9*, 90–95. https://doi.org/10.1109/mcse.2007.55.

59. Koch, T.M.I., Marks, E.S., and Roberts, T.F. (2025). A deep learning approach for the analysis of birdsong. eLife *13*, RP101111. https://doi.org/10.7554/eLife.101111.3.

60. Kuhn, H.W. (1955). The Hungarian method for the assignment problem. Nav. Res. Logist. Q. *2*, 83–97. https://doi.org/10.1002/nav.3800020109.

61. Alain, G., and Bengio, Y. (2016). Understanding intermediate layers using linear classifier probes. Preprint at arXiv. https://doi.org/10.48550/arXiv.1610.01644.

62. Li, K., Hopkins, A.K., Bau, D., Viégas, F.B., Pfister, H., and Wattenberg, M. (2023). Emergent world representations: Exploring a sequence model trained on a synthetic task. In International Conference on Learning Representations (ICLR) https://openreview.net/forum?id=DeG07_TcZvT.

63. Hewitt, J., and Manning, C.D. (2019). A Structural Probe for Finding Syntax in Word Representations. In Proceedings of the 2019 Conference of the North American Chapter of the Association for Computational Linguistics: Human Language Technologies, Volume 1 (Long and Short Papers), J. Burstein, C. Doran, and T. Solorio, eds. (Association for Computational Linguistics), pp. 4129–4138. https://doi.org/10.18653/v1/N19-1419.

64. Coenen, A., Reif, E., Yuan, A., Kim, B., Pearce, A., Viégas, F.B., and Wattenberg, M. (2019). Visualizing and measuring the geometry of BERT. Adv. Neural Inf. Process. Syst. *32*, 8594–8603.

65. Zhong, Z., and Andreas, J. (2024). Algorithmic capabilities of random transformers. In Advances in Neural Information Processing Systems (NeurIPS 2024), *20* (Curran Associates, Inc.), pp. 104357–104382. https://doi.org/10.52202/079017-3315.

66. Rahimi, A., and Recht, B. (2007). Random features for large-scale kernel machines. Adv. Neural Inf. Process. Syst. *20*, 1177–1184.

67. Rahimi, A., and Recht, B. (2009). Weighted sums of random kitchen sinks: Replacing minimization with randomization in learning. Adv. Neural Inf. Process. Syst. *21*, 1313–1320.

68. Maass, W., Natschläger, T., and Markram, H. (2002). Real-time computing without stable states: A new framework for neural computation based on perturbations. Neural Comput. *14*, 2531–2560. https://doi.org/10.1162/089976602760407955.

69. Jaeger, H. (2001). The "echo state" approach to analysing and training recurrent neural networks. GMD Report 148 (German National Research Center for Information Technology). https://doi.org/10.24406/publica-fhg-291111.

70. Cauzinille, J., Favre, B., Marxer, R., and Rey, A. (2024). Applying machine learning to primate bioacoustics: Review and perspectives. Am. J. Primatol. *86*, e23666. https://doi.org/10.1002/ajp.23666.

71. Lin, S., Gillis, W.F., Weinreb, C., Zeine, A., Jones, S.C., Robinson, E.M., Markowitz, J., and Datta, S.R. (2024). Characterizing the structure of mouse behavior using Motion Sequencing. Nat. Protoc. *19*, 3242–3291. https://doi.org/10.1038/s41596-024-01015-w.

72. von Luxburg, U. (2012). Clustering: Science or Art? JMLR Workshop Conf. SAVE Proc. *27*, 65–79.

73. Fei, Z., Fan, M., and Huang, J. (2023). A-JEPA: Joint-Embedding Predictive Architecture Can Listen. Preprint at arXiv. https://doi.org/10.48550/arXiv.2311.15830.

CellPress
OPEN ACCESS

74. Assran, M., Duval, Q., Misra, I., Bojanowski, P., Vincent, P., Rabbat, M., LeCun, Y., and Ballas, N. (2023). Self-supervised learning from images with a joint-embedding predictive architecture. In 2023 IEEE/CVF Conference on Computer Vision and Pattern Recognition (CVPR) (IEEE), pp. 15619–15629. https://doi.org/10.1109/cvpr52729.2023.01499.

75. Schneider, S., Lee, J.H., and Mathis, M.W. (2023). Learnable latent embeddings for joint behavioural and neural analysis. Nature *617*, 360–368. https://doi.org/10.1038/s41586-023-06031-6.

76. Hoffmann, J., Borgeaud, S., Mensch, A., Buchatskaya, E., Cai, T., Rutherford, E., de Las Casas, D., Hendricks, L.A., Welbl, J., Clark, A., et al. (2022). An empirical analysis of compute-optimal large language model training. In Advances in Neural Information Processing Systems (Curran Associates, Inc.), pp. 30016–30030.

77. Schulthess, L., Marty, S., Dirodi, M., Rocha, M.D., Rüttimann, L., Hahnloser, R.H.R., and Magno, M. (2023). Tinybird-ml: An ultra-low power smart sensor node for bird vocalization analysis and syllable classification. In 2023 IEEE International Symposium on Circuits and Systems (ISCAS) (IEEE), pp. 1–5.

78. Rüttimann, L., Wang, Y., Rychen, J., Tomka, T., Hörster, H., and Hahnloser, R.H.R. (2025). Multimodal system for recording individual-level behaviors in songbird groups. PeerJ *13*, e20203. https://doi.org/10.7717/peerj.20203.

79. Balsby, T.J.S., Momberg, J.V., and Dabelsteen, T. (2012). Vocal imitation in parrots allows addressing of specific individuals in a dynamic communication network. PLoS One *7*, e49747. https://doi.org/10.1371/journal.pone.0049747.

80. King, S.L., and Janik, V.M. (2013). Bottlenose dolphins can use learned vocal labels to address each other. Proc. Natl. Acad. Sci. USA *110*, 13216–13221. https://doi.org/10.1073/pnas.1304459110.

81. Sharma, P., Gero, S., Payne, R., Gruber, D.F., Rus, D., Torralba, A., and Andreas, J. (2024). Contextual and combinatorial structure in sperm whale vocalisations. Nat. Commun. *15*, 3617. https://doi.org/10.1038/s41467-024-47221-8.

82. Andreas, J., Beguš, G., Bronstein, M.M., Diamant, R., Delaney, D., Gero, S., Goldwasser, S., Gruber, D.F., de Haas, S., Malkin, P., et al. (2022). Toward understanding the communication in sperm whales. iScience *25*, 104393. https://doi.org/10.1016/j.isci.2022.104393.

83. Beguš, G., Sprouse, R., Leban, A., Silva, M., and Gero, S. (2023). Vowels and Diphthongs in Sperm Whales. Preprint at OSF. https://doi.org/10.31219/osf.io/285cs.

84. Sharma, P., Gero, S., Rus, D., Torralba, A., and Andreas, J. (2024). WhaleLM: Finding structure and information in sperm whale vocalizations and behavior with machine learning. Preprint at bioRxiv. https://doi.org/10.1101/2024.10.31.621071.

85. Vengrovski, G., Gardner, T.J., Hulsey-Vincent, M.R., and Bemrose, M. (2025). TweetyBERT. Zenodo. https://doi.org/10.5281/zenodo.15391040.

86. Cohen, Y. (2022). Song recordings and annotation files of 3 canaries used to evaluate training of TweetyNet models for birdsong segmentation and annotation. Dryad. https://doi.org/10.5061/dryad.xgxd254f4.

Patterns, Volume *7*

Supplemental information

TweetyBERT: Automated parsing of birdsong through self-supervised machine learning

George Vengrovski, Miranda R. Hulsey-Vincent, Melissa A. Bemrose, and Timothy J. Gardner

Supplementary

Layer	Sublayer	V-Measure	Dim.
3	Feed Forward Output	0.8935	196
4	Intermediate Residual Stream	0.8890	196
3	Intermediate Residual Stream	0.8859	196
3	Attention Output	0.8843	196
3	Feed Forward Output GELU	0.8829	768
2	Feed Forward Output	0.8757	196
2	Attention Output	0.8729	196
2	Feed Forward Output GELU	0.8619	768
1	Attention Output	0.8557	196
4	Attention Output	0.8494	196
4	Feed Forward Output	0.8420	196
2	Intermediate Residual Stream	0.8405	196
1	Feed Forward Output GELU	0.8400	768
1	Feed Forward Output	0.8326	196
4	Feed Forward Output GELU	0.8136	768
1	Intermediate Residual Stream	0.7518	196

Table A

Sublayer	Avg. V-Measure	Dim.
Attention Output	0.8656	196
Feed Forward Output	0.8609	196
Feed Forward Output GELU	0.8496	768
Intermediate Residual Stream	0.8418	196

Table B

Layer	Avg. V-Measure
3	0.8866
2	0.8627
4	0.8485
1	0.8201

Table C

Supplementary Table 1 | V-measure scores for layer and sublayer embeddings of TweetyBERT evaluated using UMAP projections from three birds in the TweetyNET dataset. *Scores represent averages across these test birds.* ***(A)*** *V-measure scores for each layer-sublayer combination. The selected representation (Layer 3 Attention Output) ranks fourth among all combinations; however, the difference from higher-ranked combinations is minimal and likely within measurement noise.* ***(B)*** *Crucially, Attention Output is the highest-performing sublayer averaged across all layers, and* ***(C)*** *Layer 3 provides the strongest overall representations when averaged across all sublayers, supporting its selection for downstream analyses.*

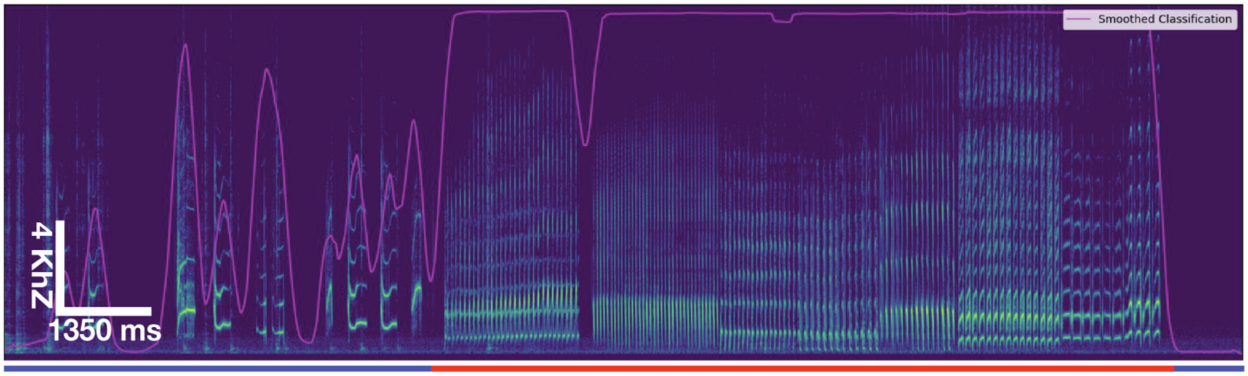

Supplementary Fig. 2 | Song Detector. *Spectrogram of a canary recording containing both song and non-song segments. The purple line represents the post-processed smoothed probabilities of each time bin being classified as canary song. The bottom bar displays the final Boolean classification of song (red) vs. non-song (blue) after post-processing. Notably, transient spikes around 1000 time bins—which are canary calls rather than song—are excluded from being labeled as song. The segmentation effectively distinguishes structured song from background noise, calls, and silent intervals, ensuring that only sustained vocal sequences are labeled as song. This figure represents a typical example of the classifier's performance across the dataset.*

The song detector used in this project is a lightweight variant of the TweetyNET architecture, designed to efficiently identify canary songs in spectrogram data. This model combines a convolutional front end with a bidirectional LSTM stack, maintaining a small hidden state size of 32 to optimize for speed and scalability. The song detector outputs a probability for each time bin, indicating the likelihood that the bin contains canary song. Ground truth labels are created by an expert human marking the start and stop of song. Spectrograms were generated using preprocessing methods previously described. During training, the model achieved a frame error rate (FER) of 2–3% on a test set containing 47 minutes of non-song and 27 minutes of song, demonstrating reliable detection of vocalizations versus background noise. The training was conducted with a learning rate of 3e-4, and the model was trained on 169 minutes of non-song and 107 minutes of song collected across a diverse set of recording conditions.

Post-processing plays a crucial role in refining the detector's output and ensuring reliable song annotations. While the raw FER is low, direct predictions can suffer from over segmentation, splitting continuous songs into fragments, or under segmentation, incorporating non-song noise into detected segments. The post-processing pipeline mitigates these issues by smoothing predictions, applying thresholds to eliminate short and noisy segments, and padding detected boundaries to ensure no valid song content is truncated. Although padding can introduce brief silences at segment edges, this tradeoff is acceptable as this has negligible performance impacts on the training of TweetyBERT.

It is important to note that the model's performance depends on the similarity between the training and deployment environments. Since the training data was manually annotated and collected in a consistent seasonal period and recording setup, the detector performs well within this context. However, deployment in different conditions may require retraining or fine-tuning to maintain accuracy.

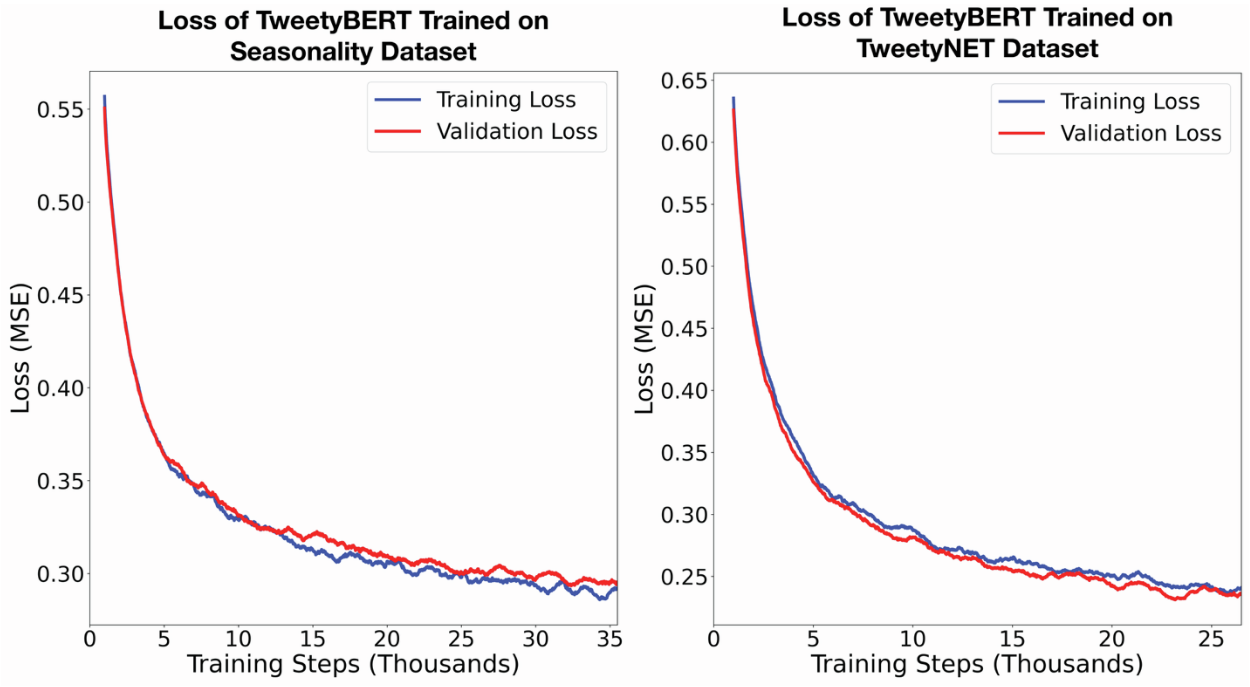

Supplementary Fig. 3 | TweetyBERT MSE Loss vs Training Steps. *Training and validation loss curves (mean squared error, MSE) for TweetyBERT trained separately on the Seasonality and TweetyNET datasets. Both training (blue) and validation (red) losses exhibit stable convergence, indicating effective self-supervised learning of masked spectrogram reconstructions. The higher final MSE observed for the Seasonality dataset likely reflects greater acoustic variability between breeding and non-breeding vocalizations, consistent with biologically driven seasonal vocal plasticity.*

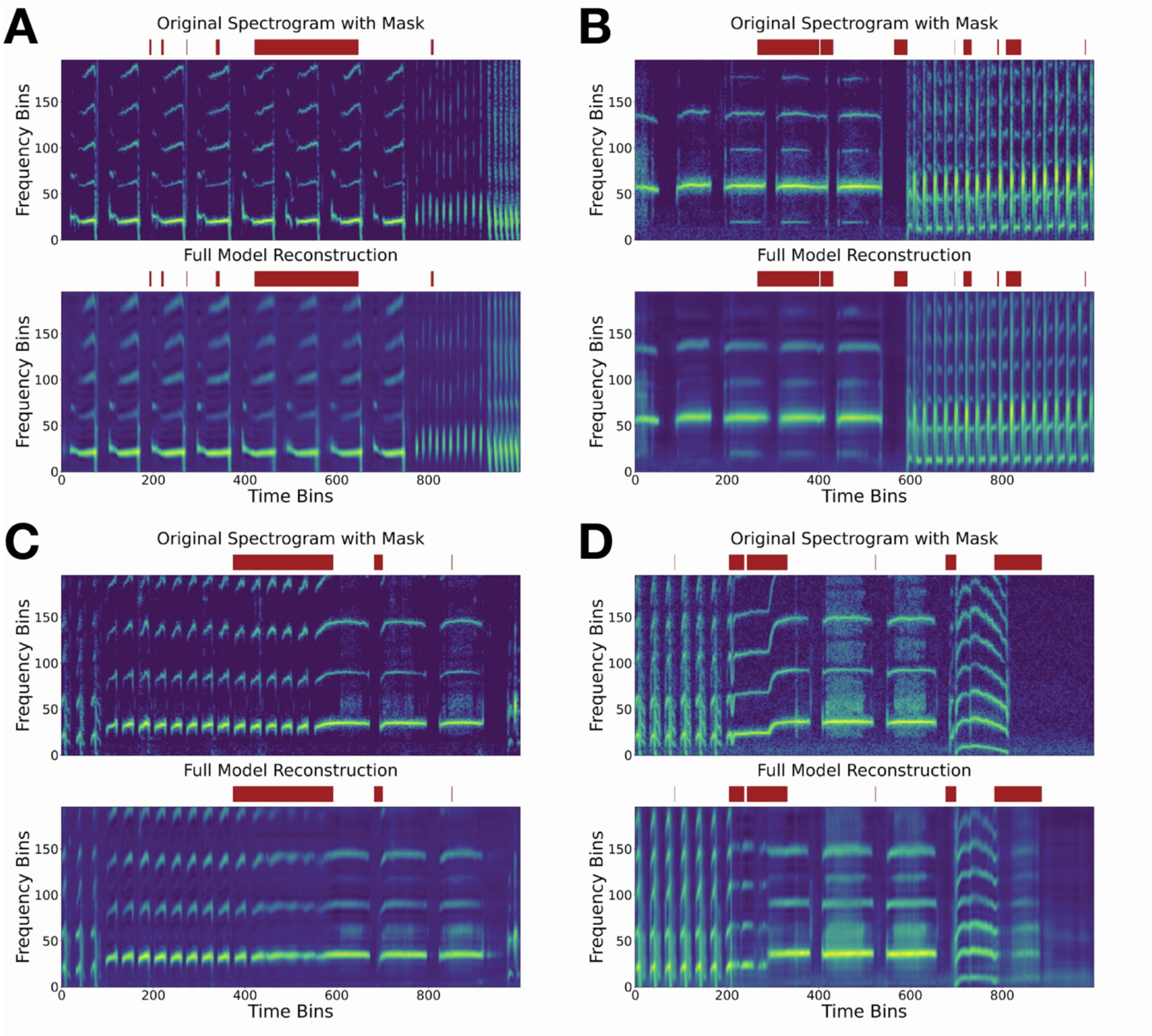

Supplementary Fig. 4 | Montage of masked spectrogram predictions from the TweetyBERT training phase. *Each panel consists of a masked spectrogram fed to TweetyBERT (top) and the model's predicted reconstruction (bottom). Red bars indicate masked regions that were not visible to the model during this prediction.* ***(A, B)*** *Depict accurate reconstructions of masked segments, demonstrating the model's capability to infer detailed acoustic structures from context.* ***(C, D)*** *Illustrate mild reconstruction errors where the model produces hybrid syllable morphologies by combining two syllable classes. These errors arise when critical contextual cues are obscured by masking, causing ambiguity in syllable prediction.*

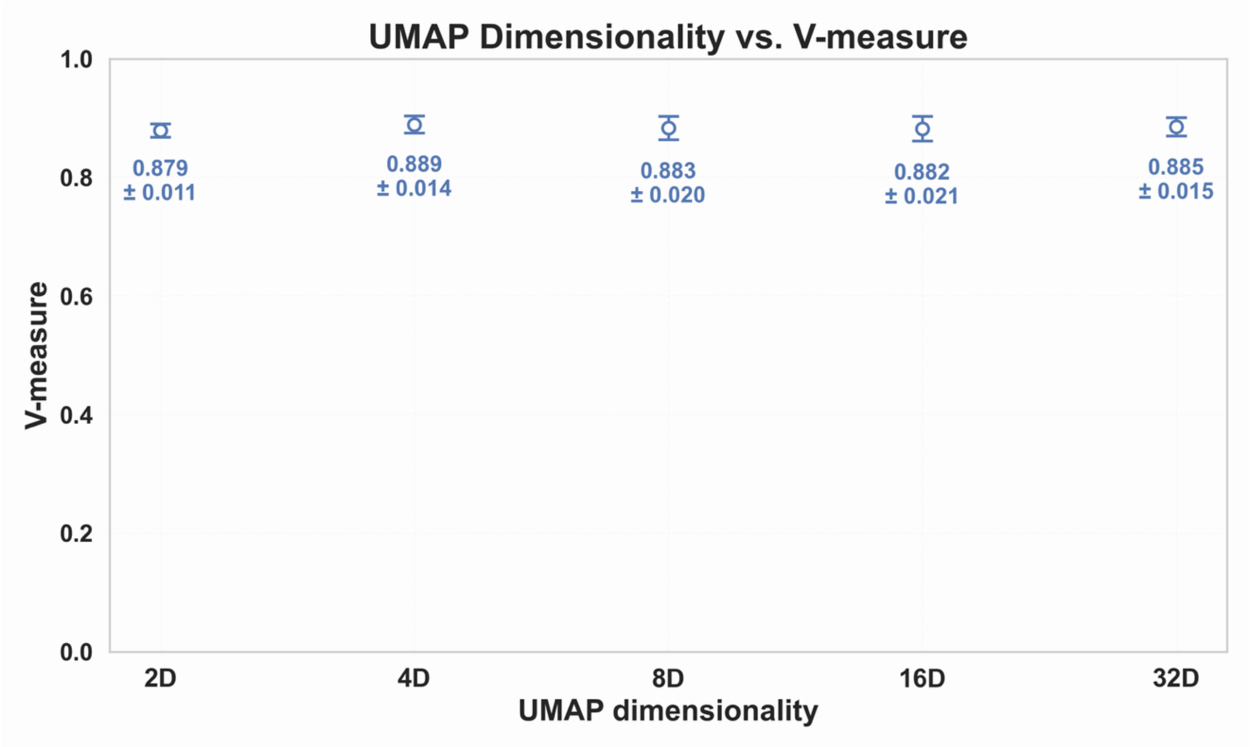

Supplementary Fig. 5 | V-Measure score as a function of UMAP Dimensionality. *The points summarize V-measure clustering scores obtained across 12 held-out folds of TweetyBERT latent representations. Boxes mark the standard deviation, and the filled circles indicate the fold-wise mean. It is apparent that the relationship between UMAP dimensionality and V-Measure score is weak.*

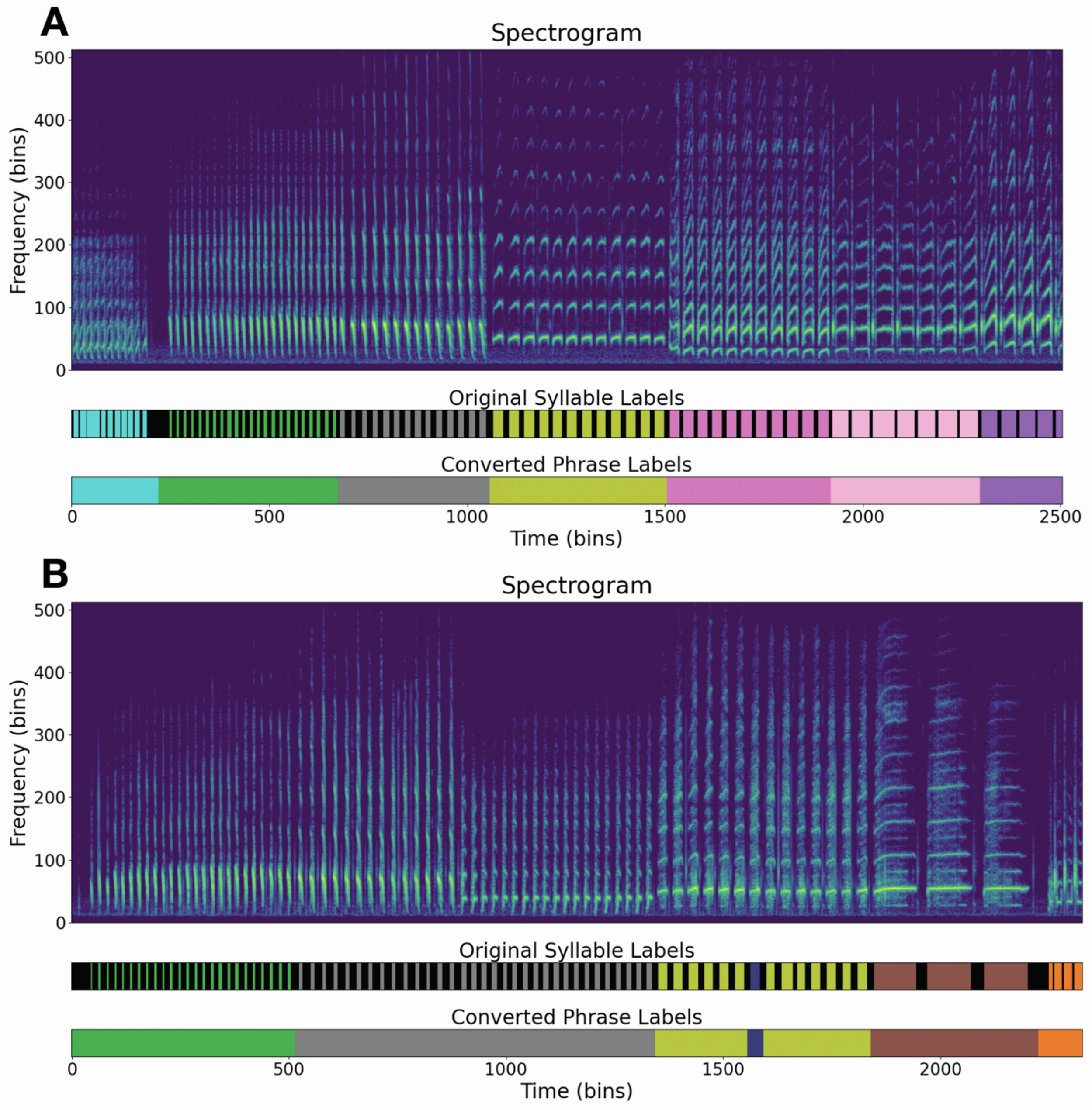

Supplementary Fig. 6 | Examples of conversion from syllable-level to phrase-level labels. ***(A, B)*** *Spectrograms of complete canary songs, each shown with original ground truth syllable-level annotations (top, silences in black) and their corresponding phrase-level labels after conversion (bottom). During conversion, silent intervals between syllables were reassigned to their nearest neighboring syllable labels. Panel* ***(B)*** *highlights two annotation errors in the original ground truth labels: a mislabeled syllable insertion (purple within yellow) and two distinct phrases incorrectly labeled as identical (gray).*

Smoothing Window (Time bins)	Phrase Entropy Correlation (r)	Phrase Duration Correlation (r)	V-Measure	FER (mapped)	FER (mapped and unmapped)
0	0.337	0.686	0.868	7.74%	15.98%
25	0.615	0.868	0.872	7.28%	15.59%
50	0.658	0.904	0.882	6.76%	15.12%
75	0.710	0.928	0.897	7.04%	15.73%
100	0.702	0.931	0.899	6.61%	15.65%
125	0.713	0.940	0.898	5.76%	14.92%
150	0.745	0.947	0.893	4.97%	14.51%
175	0.749	0.950	0.891	4.99%	14.52%
**200**	**0.771**	**0.957**	**0.888**	**4.34%**	**13.97%**
225	0.778	0.960	0.885	4.44%	14.06%
250	0.772	0.959	0.881	4.58%	14.18%
275	0.769	0.960	0.878	4.70%	14.29%
300	0.761	0.959	0.874	4.84%	14.42%
325	0.750	0.958	0.871	5.00%	14.53%
350	0.742	0.960	0.866	5.06%	14.66%
375	0.738	0.960	0.863	5.20%	14.78%
400	0.736	0.957	0.858	5.46%	14.94%
425	0.723	0.943	0.855	6.90%	16.24%
450	0.723	0.943	0.851	7.05%	16.42%
475	0.715	0.941	0.847	7.29%	16.63%
500	0.716	0.941	0.843	7.46%	16.87%

Supplementary Table 7 | Raw data evaluating the impact of smoothing window size on clustering performance. *Pearson correlations between HDBSCAN-derived and ground truth measures of phrase entropy and phrase duration, V-measure scores, and Frame Error Rates.*

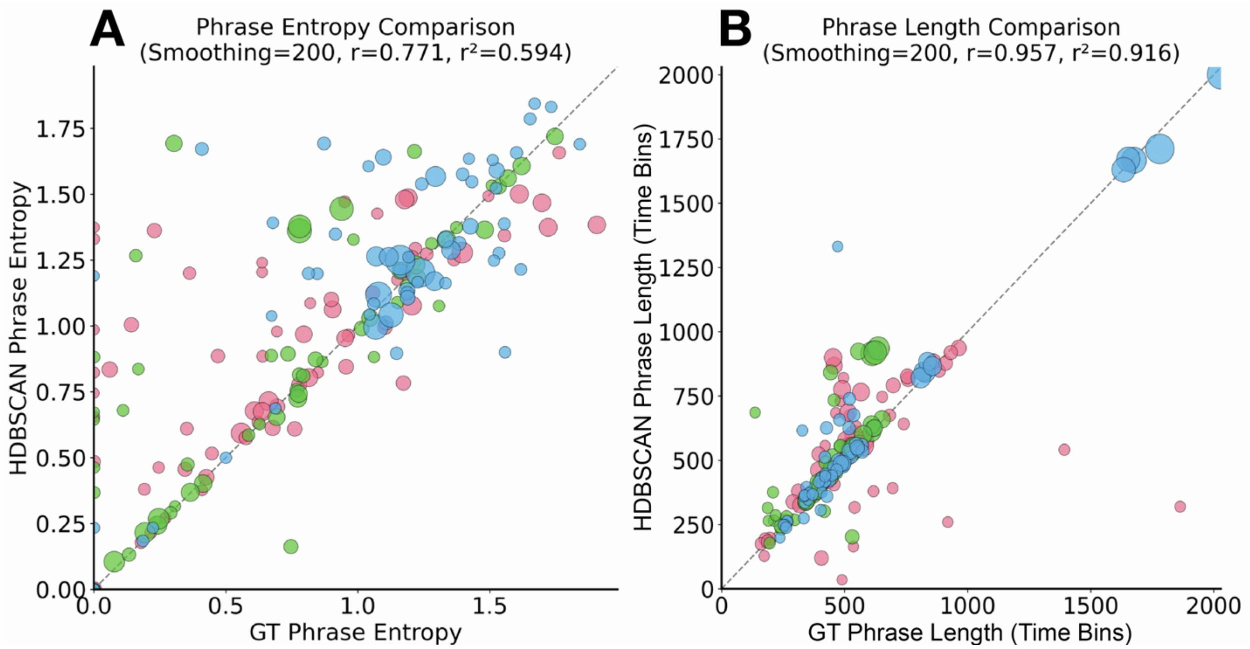

Supplementary Fig. 8 | Phrase entropy correlations for each individual phrase label across three birds. *Relationship between TweetyBERT-derived phrase metrics and human-derived ground truth (GT) metrics for three birds (colors represent individual birds).* ***(A)*** *Phrase entropy correlations* ***(B)*** *Phrase duration correlations. All analysis shown here were conducted with a 200 time bin smoothing window.*
